# Mortality and concurrent use of opioids and hypnotics in older patients: A retrospective cohort study

**DOI:** 10.1371/journal.pmed.1003709

**Published:** 2021-07-15

**Authors:** Wayne A. Ray, Cecilia P. Chung, Katherine T. Murray, Beth A. Malow, James R. Daugherty, C. Michael Stein

**Affiliations:** 1 Department of Health Policy, Vanderbilt University School of Medicine, Nashville, Tennessee, United States of America; 2 Cecilia P. Chung, Department of Medicine, Vanderbilt University School of Medicine, Nashville, Tennessee, United States of America; 3 Departments of Medicine and Pharmacology, Vanderbilt University School of Medicine, Nashville, Tennessee, United States of America; 4 Department of Neurology, Vanderbilt University School of Medicine, Nashville, Tennessee, United States of America; Universitetet i Oslo, NORWAY

## Abstract

**Background:**

Benzodiazepine hypnotics and the related nonbenzodiazepine hypnotics (z-drugs) are among the most frequently prescribed medications for older adults. Both can depress respiration, which could have fatal cardiorespiratory effects, particularly among patients with concurrent opioid use. Trazodone, frequently prescribed in low doses for insomnia, has minimal respiratory effects, and, consequently, may be a safer hypnotic for older patients. Thus, for patients beginning treatment with benzodiazepine hypnotics or z-drugs, we compared deaths during periods of current hypnotic use, without or with concurrent opioids, to those for comparable patients receiving trazodone in doses up to 100 mg.

**Methods and findings:**

The retrospective cohort study in the United States included 400,924 Medicare beneficiaries 65 years of age or older without severe illness or evidence of substance use disorder initiating study hypnotic therapy from January 2014 through September 2015. Study endpoints were out-of-hospital (primary) and total mortality. Hazard ratios (HRs) were adjusted for demographic characteristics, psychiatric and neurologic disorders, cardiovascular and renal conditions, respiratory diseases, pain-related diagnoses and medications, measures of frailty, and medical care utilization in a time-dependent propensity score–stratified analysis. Patients without concurrent opioids had 32,388 person-years of current use, 260 (8.0/1,000 person-years) out-of-hospital and 418 (12.9/1,000) total deaths for benzodiazepines; 26,497 person-years,150 (5.7/1,000) out-of-hospital and 227 (8.6/1,000) total deaths for z-drugs; and 16,177 person-years,156 (9.6/1,000) out-of-hospital and 256 (15.8/1,000) total deaths for trazodone. Out-of-hospital and total mortality for benzodiazepines (respective HRs: 0.99 [95% confidence interval, 0.81 to 1.22, *p* = 0.954] and 0.95 [0.82 to 1.14, *p* = 0.513] and z-drugs (HRs: 0.96 [0.76 to 1.23], *p* = 0.767 and 0.87 [0.72 to 1.05], *p* = 0.153) did not differ significantly from that for trazodone. Patients with concurrent opioids had 4,278 person-years of current use, 90 (21.0/1,000) out-of-hospital and 127 (29.7/1,000) total deaths for benzodiazepines; 3,541 person-years, 40 (11.3/1,000) out-of-hospital and 64 (18.1/1,000) total deaths for z-drugs; and 2,347 person-years, 19 (8.1/1,000) out-of-hospital and 36 (15.3/1,000) total deaths for trazodone. Out-of-hospital and total mortality for benzodiazepines (HRs: 3.02 [1.83 to 4.97], *p* < 0.001 and 2.21 [1.52 to 3.20], *p* < 0.001) and z-drugs (HRs: 1.98 [1.14 to 3.44], *p* = 0.015 and 1.65 [1.09 to 2.49], *p* = 0.018) were significantly increased relative to trazodone; findings were similar with exclusion of overdose deaths or restriction to those with cardiovascular causes. Limitations included composition of the study cohort and potential confounding by unmeasured variables.

**Conclusions:**

In US Medicare beneficiaries 65 years of age or older without concurrent opioids who initiated treatment with benzodiazepine hypnotics, z-drugs, or low-dose trazodone, study hypnotics were not associated with mortality. With concurrent opioids, benzodiazepines and z-drugs were associated with increased out-of-hospital and total mortality. These findings indicate that the dangers of benzodiazepine–opioid coadministration go beyond the documented association with overdose death and suggest that in combination with opioids, the z-drugs may be more hazardous than previously thought.

## Abstract 2

In a retrospective cohort study, Dr. Wayne Ray and colleagues investigate concurrent opioid and hypnotic use and mortality in older adults in US.

## Author summary

**Why was this study done?:**

- Benzodiazepines and the related z-drugs are sleep medications commonly prescribed for persons 65 years of age or older.
- Both can interfere with breathing, which, in turn, may cause irregular heartbeat and deaths.
- Opioid painkillers, often used with sleep medications, also interfere with breathing and thus could increase the likelihood of deaths with benzodiazepines or z-drugs.
- Trazodone, an antidepressant that in low doses often is prescribed for insomnia, has not been found to affect breathing and thus may be safer for older patients, particularly for those also taking opioids.

**What did the researchers do and find?:**

- We identified 400,924 persons in the US Medicare program 65 or older who began treatment with benzodiazepine, z-drug, or trazodone sleep medications.
- For patients without and with opioid use, we compared the rate of out-of-hospital deaths—often related to heart problems—and total mortality during treatment with benzodiazepines and z-drugs to that for trazodone.
- For patients with opioid exposure, benzodiazepines were associated with respective 302% and 221% increases in out-of-hospital and total mortality, and z-drugs were associated with respective 98% and 65% increases in out-of-hospital and total mortality.

**What do these findings mean?:**

- In older populations, the risks of concurrent benzodiazepine–opioid exposure go beyond the known increased likelihood of overdose, suggesting that greater efforts are needed to prevent use of this medication combination.
- Patients and clinicians should be aware that in combination with opioids, the z-drugs, thought to be relatively safe, may increase the risk of death.

## Introduction

Hypnotics are among the most frequently prescribed medications for older adults [1–3]. The 3 most commonly prescribed medications for insomnia in the US are the benzodiazepines, the z-drugs, and low-dose trazodone [4,5]. Benzodiazepines stimulate γ-aminobutyric acid type A (GABA-A) receptors, the major inhibitory neurotransmitter in the central nervous system [6]. The z-drugs (zolpidem and related nonbenzodiazepine hypnotics) are GABA-A agonists that are more selective for the α1 subtype receptors thought to mediate hypnotic effects [7] and thus hypothesized to reduce the risk of dependence, psychomotor impairment, and injuries reported for benzodiazepines [6,8–10]. Trazodone, an antidepressant without GABA activity [4,11–13], in low doses is often prescribed for insomnia [4,5], although it is not labeled as a hypnotic and guidelines generally only recommend use for insomnia associated with a mood disorder [14].

Although the primary concern with benzodiazepines and z-drugs has been the risk of dependence and injuries [6,8–10], these drugs also have potentially fatal respiratory effects. Benzodiazepines depress respiration [7,15,16] and thus can exacerbate sleep-disordered breathing [17,18], which, in turn, can trigger life-threatening cardiac arrhythmias [19–21]. In overdose, benzodiazepines can cause airway obstruction or respiratory failure [22]. The z-drugs also impair respiration, although to a lesser degree than for benzodiazepines [18,23]. In contrast, respiratory impairment has not been observed for trazodone [24,25], although it infrequently has serious adverse cardiovascular effects [26,27].

The frequent use of opioid analgesics with both benzodiazepines and z-drugs [28–31] could increase the risk of adverse cardiorespiratory effects. Opioids profoundly depress respiration [32–34], cause sleep-disordered breathing [34, 35], increase the likelihood of airway obstruction and respiratory failure [36], and are associated with increased risk of out-of-hospital cardiovascular death [37]. Although the US Food and Drug Administration (FDA) requires a boxed warning (“black box”) concerning coadministration of benzodiazepines and opioids because of greater likelihood of overdose death [28], such deaths are relatively uncommon in older populations [38]. However, an increased risk of cardiovascular deaths, the most common cause of death for persons 65 years of age or older [38], with concurrent use of benzodiazepines or z-drugs with opioids would have major public health implications.

Thus, to better define the relative safety of commonly prescribed hypnotics, both without and with concurrent opioids, we compared mortality in US Medicare enrollees 65 years of age or older without severe illnesses initiating treatment with benzodiazepine hypnotics, z-drugs, or low-dose trazodone.

## Methods

### Design

We conducted a retrospective cohort study using the computerized files of the US Medicare program, which provided an efficient means to identify the cohort and obtain study data [39]. The protocol is shown in S1 Protocol. The study is reported as per the Strengthening the Reporting of Observational Studies in Epidemiology (STROBE) guideline (S1 Checklist). The study was approved by the Vanderbilt Medical Center Ethics Committee.

### Cohort

#### Medicare data

Medicare provides healthcare insurance for nearly all US citizens 65 years of age or older [40]. All Medicare beneficiaries have coverage for inpatient/skilled nursing facility stays (Part A) and also may elect coverage for outpatient services (Part B) and prescription medications (Part D). Enrollees can choose either a fee-for-service plan or Medicare Advantage (Part C), a managed care model. To ensure completeness of data, our study was restricted to enrollees with Parts A, B, and D who had fee-for-service coverage, because Medicare Advantage healthcare encounter data were considered less reliable during the study years [40]. In 2019, there were 53 million Medicare enrollees 65 years of age or older, of whom 26 million had fee-for-service Parts A, B, and D [41].

The Medicare enrollment file records periods of enrollment for each part of Medicare. It identifies deaths for >95% of persons 65 and older in the US [40], which have been linked to the National Death Index (NDI). Other files include medical care encounters for pharmacy, hospital, outpatient, and nursing home services. The data reside in the Center for Medicare & Medicaid Services (CMS) Chronic Condition Warehouse (Section C.1 in S1 Appendix) and were accessed through the Virtual Research Data Center (VRDC), a cloud-based repository of de-identified Medicare files [42]. In accordance with CMS policy, no table cells with fewer than 11 patients were reported. The study was approved by the Vanderbilt University Medical Center Institutional Review Board, with waiver of informed consent.

#### Study medications

Study hypnotics (Table A in S1 Appendix) included selected benzodiazepines, z-drugs, and trazodone in doses up to 100 mg (recommended for hypnotic use, but lower than doses for treatment of mood disorders) [12]. The study benzodiazepines were those with a labeled hypnotic indication (estazolam, flurazepam, quazepam, temazepam, and triazolam), as well as alprazolam, clonazepam, and lorazepam, which have similar pharmacodynamic properties, are prescribed for insomnia and are included in guidelines for hypnotics [6,10]. Patients for whom the prescribed regimen was inconsistent with hypnotic use (more than 1 tablet/capsule per day) or for whom there was evidence of an alternative indication for benzodiazepines or trazodone (diagnosis in the past 90 days indicating panic disorder, anxiety/post-traumatic stress disorder, neurologic indications for benzodiazepines, major depression, or bipolar disorder) were excluded from the cohort (Section A in S1 Appendix).

The study opioids (Table B in S1 Appendix) excluded parenteral opioids (infrequently prescribed for outpatients) and preparations specifically formulated for cough or diarrhea. Opioids were classified as short or long acting, and dose equivalents were calculated in morphine milligram equivalents (MME) according to guidelines for chronic opioid therapy for non-cancer pain [43].

#### Cohort eligibility

The cohort (Table C in S1 Appendix) consisted of Medicare beneficiaries 65 years of age or older who filled a study hypnotic prescription between January 1, 2014 and September 29, 2015. They had to have complete demographic information and in the past year have enrollment in Medicare Parts A, B, and D but not Part C, thus limiting the cohort to beneficiaries with fee-for-service coverage. To assure regular contact with medical care, cohort members had to have at least 1 outpatient visit and 1 filled prescription (other than the study hypnotic) in the prior year.

The cohort was restricted to new users of hypnotic medications, which permits better ascertainment of deaths early in therapy in susceptible patients and assures baseline covariates are not influenced by the study hypnotics [44]. Thus, cohort members could not have filled a prescription in the past year for any study hypnotic (other than a single day of supply, often periprocedural), non-study benzodiazepine, or non-study hypnotic (Table C in S1 Appendix).

Hypnotics and opioids often are initiated for patients with unstable or life-threatening illness, which increases the likelihood of difficult-to-control confounding [6,45] and reduces the capacity to detect drug-associated mortality [37]. Thus, the cohort excluded patients in nursing homes, with hospice care, with recent hospitalizations indicating unstable illness, and with active cancer or other life-threatening disease (Table D in S1 Appendix). To assess the effects of therapeutic study drug use, patients with diagnosed substance use disorder or use of buprenorphine, most commonly prescribed as opioid replacement therapy, were excluded.

#### Follow-up

Cohort follow-up began on the day following the fill date for the first study hypnotic prescription. Follow-up ended with prescription of a hypnotic medication from a different class, a non-study benzodiazepine or trazodone >100 mg as well as with loss of full fee-for-service (Parts A, B, and D and no C) Medicare enrollment, nursing home or hospice entry, hospital stay >30 days, substance use disorder diagnosis, September 30, 2015, or death. Patients who left the cohort could not reenter. Because the postulated mechanisms by which study medications affect mortality require presence of the drug [17], follow-up and analysis were restricted to current hypnotic use (Fig A in S1 Appendix): the prescription fill through end of dispensed days of supply (offset 1 day to account for probable nocturnal hypnotic use).

**Fig 1:**
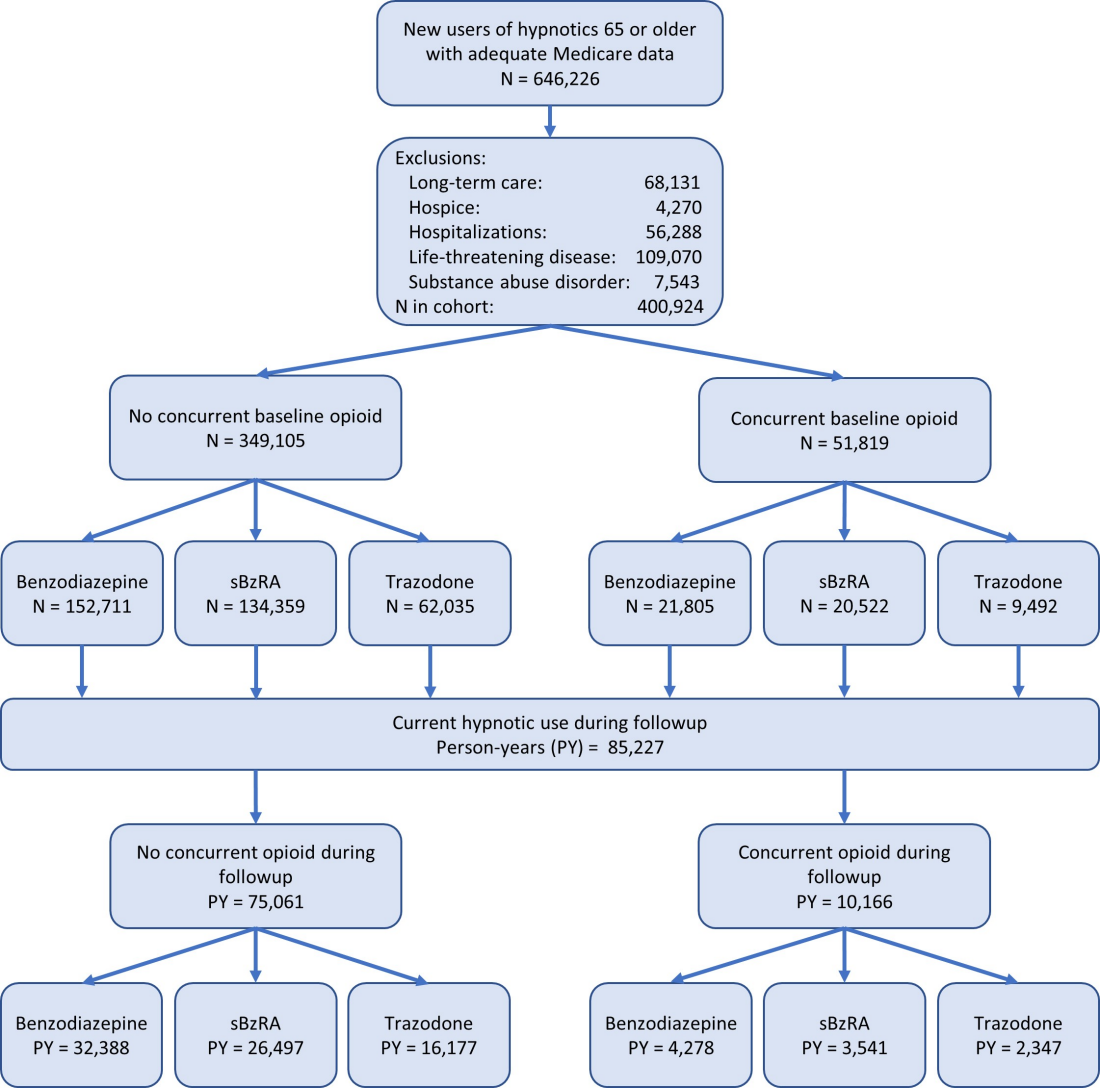
Study design. *N* denotes number of patients. PY, person-year.

Opioids could be started or stopped at any time during follow-up. Thus, both current use and its characteristics (start past 90 days, long-acting drug, dose) were determined for each person-day of follow-up. A single patient could have person-time both with and without concurrent opioids (Fig 1); however, because there was no overlap and the endpoint occurred only once, statistical independence assumptions were satisfied [24].

### Endpoints

Deaths were identified from the Medicare Master Beneficiary Summary File, and the underlying cause of death (Table E in S1 Appendix) came from the linked NDI. The small number of beneficiaries with a Medicare date of death but no linked NDI record (1.4%) were considered deaths of unknown cause.

The primary endpoint included all out-of-hospital deaths for several reasons. First, in patients without severe illness, a substantial proportion of deaths are plausibly related to cardiac arrhythmias. In a previous investigation of opioid use for non-cancer chronic pain with medical record review [37,46], 73% of out-of-hospital deaths were sudden unexpected deaths consistent with a cardiac arrhythmia. Second, both our experience [37] and that of others [47] suggests that US death certificates under-ascertain cardiovascular deaths, particularly for out-of-hospital deaths in older patients for whom postmortem investigations may be limited [48]. Third, benzodiazepines and opioids, particularly in overdose, have respiratory effects—decreased ventilation, airway obstruction, and respiratory failure [22,36]—that can lead to sudden unexpected death. A sensitivity analysis restricted deaths to those with a cardiovascular cause.

### Analysis

#### Covariates

Study comparisons controlled for 105 covariates plausibly associated with both risk of death and the use of specific hypnotics or opioids (Section G and Table F in S1 Appendix). The covariates, defined from medical care encounters in the year preceding cohort entry, were based on our previous studies of out-of-hospital death [37,46], standard measures of comorbidity [49,50], and indicators of frailty [51]. They included demographic characteristics (including self-reported race, indicator of genetic factors potentially influencing drug metabolism), psychiatric and neurologic disorders, cardiovascular and renal conditions, respiratory diseases, pain-related diagnoses and medications, measures of frailty, and medical care utilization. Because comorbidity could change during follow-up, covariate values (other than opioid characteristics) were updated on the date of each hypnotic prescription fill.

#### Propensity score

All covariates (other than opioid characteristics) were controlled for by stratifying the analysis according to deciles (distribution in trazodone group) of the appropriate pairwise propensity score, the probability of trazodone use, given the covariates (Section H in S1 Appendix) [52,53]. Because the covariates were updated at the time of each hypnotic prescription fill during follow-up, the propensity scores were time dependent [54]. After weighting, the distributions of covariates differed little between treatment groups (Table G in S1 Appendix).

#### Statistical analysis

The adjusted relative risk of death was estimated with hazard ratios (HRs) from a stratified time-dependent proportional hazards regression (Section I in S1 Appendix) [55]. The timescale was cumulative days of current hypnotic use, creating risk sets of patients with the same treatment duration. For patients with opioid use, the model included terms for opioid current use and its characteristics. Because opioid prescriptions could be filled at any time during follow-up, these variables were updated on a daily basis. Statistical significance was defined as 95% Wald confidence intervals that excluded 1, with *p*-values calculated accordingly. Because the study comparisons were planned, *p*-values were not adjusted for multiple comparisons. All analyses were done with SAS version 9.4.

#### Sensitivity analyses

Sensitivity analyses were performed that examined groups of particular interest or used an alternative statistical analysis. The former included restriction of endpoints to non-overdose deaths or deaths from cardiovascular causes and exclusion of possible non-hypnotic benzodiazepine use by requiring the benzodiazepine to be labeled as a hypnotic or the patient to have a recent insomnia diagnosis. The alternative statistical analyses (Section I in S1 Appendix) accounted for geographic region (assessed region both as clustering factor and confounder), fixed all covariates at baseline except for opioid use and characteristics (guards against causal pathway confounding), had time-dependent propensity score weights based on all covariates (residual confounding), and used baseline pairwise propensity score matching with only opioid covariates time dependent (causal pathway and residual confounding).

## Results

### Characteristics of cohort at baseline

#### Entire cohort

There were 646,226 new users of study hypnotic medications 65 years of age or older with adequate Medicare data, of whom 400,924 qualified for the cohort (Fig 1). The most frequently prescribed individual benzodiazepines were alprazolam (39.8% of patients receiving benzodiazepine), lorazepam (27.7%), and temazepam (17.9%); zolpidem was the most frequently prescribed z-drug (91.1%). Cohort members had a mean age of 75.5 (std 7.3) years, and 65.9% were female.

#### No concurrent opioid

At baseline, 349,105 cohort members did not have concurrent opioid use, including 152,711 new users of benzodiazepines, 134,359 of z-drugs, and 62,035 of trazodone. Comorbidity levels in the past year were consistently higher for the trazodone group than for z-drugs (Table 1, Table F in S1 Appendix). The patients beginning trazodone use were more likely to have Medicaid enrollment (22.4% versus 14.7%), mood disorders (19.7% versus 10.3%), dementia (13.5% versus 3.9%), chronic kidney disease (12.6% versus 9.1%), chronic obstructive pulmonary disease (13.1% versus 10.1%), history of falls (9.2% versus 5.5%), and malnutrition or feeding problems (8.9% versus 6.2%). The comorbidity levels for patients initiating trazodone were also higher than those for benzodiazepines, although the differences were less pronounced.

**Table 1:** Baseline cohort characteristics^a^ according to concurrent opioid use.

	No concurrent opioid use			Concurrent opioid use		
	Benzodiazepines	z-Drugs	Trazodone	Benzodiazepines	z-Drugs	Trazodone
*N*	152,711	134,359	62,035	21,805	20,522	9,492
Dose, mean, mg^b^	8.9	7.4	52.2	10.0	7.6	54.9
Age, mean (std)	76.1 (7.6)	74.3 (6.8)	76.6 (7.8)	75.6 (7.6)	74.0 (6.8)	75.6 (7.7)
Female	70.3%	60.4%	67.5%	68.8%	60.2%	67.2%
White race^c^	87.2%	84.9%	82.6%	85.5%	84.4%	81.5%
Entered cohort in 2014	57.6%	59.0%	54.1%	58.3%	59.4%	56.5%
Medicaid	15.7%	14.7%	22.4%	25.5%	23.5%	35.3%
*Psychiatric/neurologic*						
Mood disorder^d^	13.5%	10.1%	19.4%	16.6%	13.6%	23.0%
Anxiety, panic disorder, or PTSD^e^	8.7%	4.3%	5.8%	8.6%	5.2%	6.9%
Alzheimer and other dementias	8.3%	3.8%	13.4%	6.3%	3.9%	8.7%
Selective serotonin reuptake inhibitors	19.3%	14.1%	20.0%	20.8%	17.5%	22.8%
Other antidepressant	10.4%	9.6%	14.2%	17.2%	16.1%	22.2%
*Cardiovascular/renal*						
Myocardial infarction	3.9%	3.5%	4.1%	4.9%	5.1%	5.3%
Heart failure	8.8%	7.6%	9.6%	12.2%	10.4%	13.2%
Diabetes	30.8%	29.4%	33.0%	36.5%	35.8%	38.1%
Smoking and smoking-related disorders	9.4%	9.4%	11.8%	14.7%	16.0%	19.0%
Chronic kidney disease	10.5%	9.0%	12.6%	13.5%	12.1%	16.0%
Beta-blockers	39.9%	34.6%	38.9%	42.0%	38.2%	44.0%
Diuretics, loop	12.8%	10.5%	14.7%	20.9%	17.3%	23.5%
Hypoglycemics, insulin	4.7%	4.5%	6.5%	7.6%	7.2%	9.5%
Hypoglycemics, metformin	13.5%	14.0%	16.6%	16.7%	17.1%	18.0%
*Respiratory*						
Chronic obstructive pulmonary disease	11.7%	10.0%	12.8%	18.2%	16.2%	21.4%
Home oxygen	4.3%	4.0%	5.0%	7.2%	6.1%	8.6%
Beta-agonists	11.5%	11.2%	12.7%	16.0%	15.6%	19.0%
*Pain*						
Neuropathic pain	21.2%	21.5%	20.7%	36.7%	36.5%	36.8%
Headache, including migraine	10.2%	8.9%	10.2%	12.6%	11.6%	12.5%
Cyclobenzaprine/other skeletal muscle relaxant	6.3%	6.5%	6.8%	15.9%	16.3%	18.2%
Gabapentinoids/carbamazepine	9.8%	9.5%	11.6%	23.6%	22.8%	28.8%
*Frailty*						
Unintentional fall (not vigorous activity)	7.0%	5.4%	9.1%	10.5%	9.2%	12.3%
Wheelchair, hospital bed, or difficulty transfers	2.3%	1.8%	3.3%	4.8%	4.0%	6.1%
Malnutrition^f^	7.4%	6.1%	8.8%	9.1%	7.6%	10.7%
*Medical care in past 90 days*						
Inpatient discharge	1.8%	2.2%	2.3%	3.1%	4.6%	3.7%
Emergency department visit	9.9%	7.4%	10.9%	15.1%	13.0%	15.4%
Home health visit	4.3%	3.0%	5.6%	7.8%	6.7%	9.1%
*Opioid characteristics*						
Long acting	0.0%	0.0%	0.0%	4.8%	4.3%	5.7%
Started past 90 days	0.0%	0.0%	0.0%	25.1%	27.1%	13.9%
Dose ≥60 morphine milligram equivalents	0.0%	0.0%	0.0%	29.8%	30.4%	34.6%

Unless otherwise specified, covariates that are based on medical care reflect encounters in the year prior to cohort entry.

^a^Some important covariates selected by the authors as examples of the study covariates; see Table F in S1 Appendix for the complete set.

^b^Benzodiazepines: diazepam equivalents; z-drugs: zolpidem equivalents.

^c^Race is self-reported to Medicare.

^d^Cohort excludes patients with diagnosis of major depression or bipolar disorder in past 90 days.

^e^Cohort excludes patients with a diagnosis of panic or anxiety disorder or PTSD in past 90 days.

^f^Includes diagnosis of malnutrition, abnormal weight loss, feeding problems, and dysphagia.

PTSD, post-traumatic stress disorder.

#### Concurrent opioid

At baseline, 51,819 cohort members had concurrent opioid use, 12.9% of the cohort. These included 21,805 new users of benzodiazepines, 20,522 of z-drugs, and 9,492 of trazodone. The covariates indicated greater comorbidity for the trazodone group than for the z-drugs (Table 1, Table F in S1 Appendix) as well as similar, although less pronounced, differences between patients receiving trazodone and benzodiazepines. Those with use of trazodone were less likely to have started the opioid within the past 90 days but were more likely to receive a long-acting drug and higher doses.

### Deaths during follow-up

#### Entire cohort

During 85,277 person-years of follow-up, there were 1,128 deaths (13.2/1,000 person-years), of which 715 (8.4/1,000) were out-of-hospital, and 413 (4.8/1,000) were in-hospital. The underlying causes of study deaths were cardiovascular (59.0% of deaths), respiratory (10.2%), neurologic (6.9%), injuries (6.8%), cancer (4.3%), and other causes (12.8%).

#### No concurrent opioid

**Fig 2:**
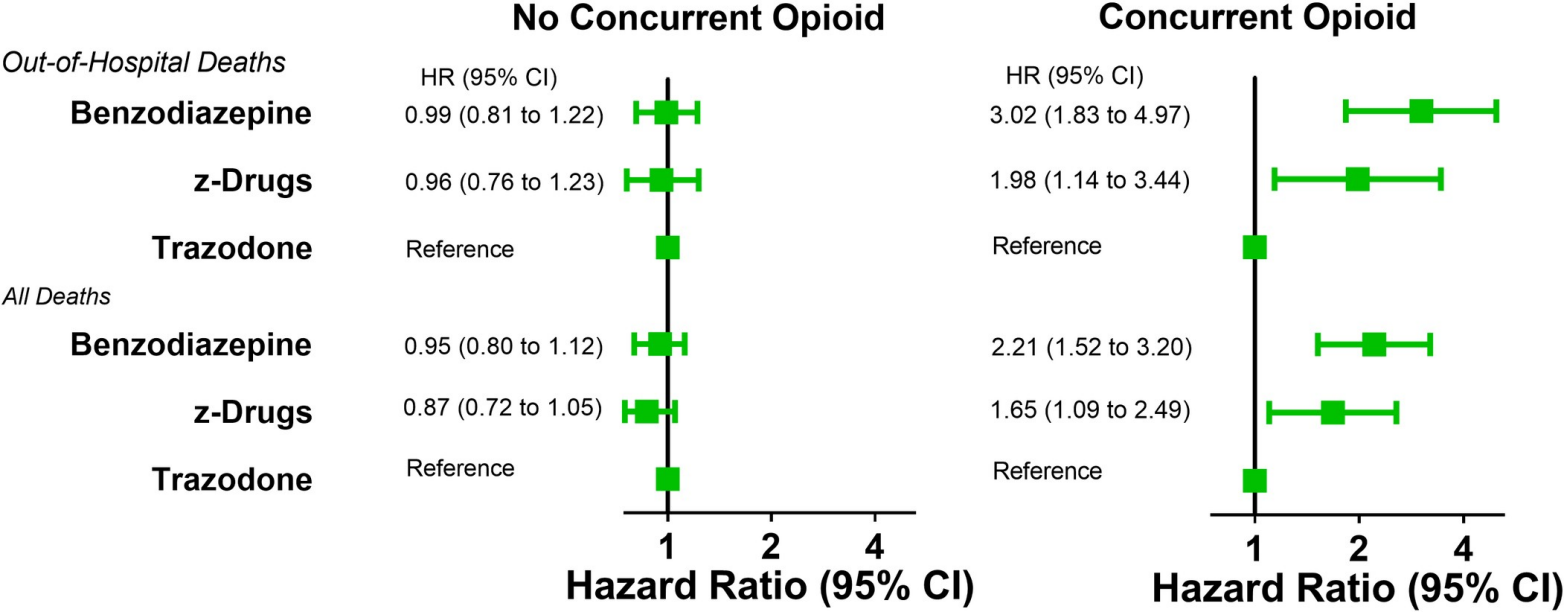
Adjusted HRs for benzodiazepines and z-drugs vs. trazodone for out-of-hospital and total mortality, according to concurrent opioid use. The bars indicate the 95% confidence intervals for the HRs. The adjusted HRs for the comparisons of benzodiazepines vs. z-drugs are shown in Table H in S1 Appendix. HR, hazard ratio.

During 75,061 person-years of follow-up without concurrent opioid use, there were 32,388 person-years and 260 out-of-hospital deaths for benzodiazepines (8.0/1,000), 26,497 person-years and 150 deaths for z-drugs (5.7/1,000), and 16,177 person-years and 156 deaths (9.6/1,000) for trazodone (Table 2). After adjustment for covariates, the risk of out-of-hospital death for benzodiazepines (Fig 2, HR = 0.99 [0.81 to 1.23], *p* = 0.954) and z-drugs (HR = 0.96 [0.76 to 1.23], *p* = 0.767) did not differ significantly from that for trazodone, nor were there significant differences in the adjusted risk of total study mortality (benzodiazepines, HR = 0.95 [0.80 to 1.12], *p* = 0.513; z-drugs, HR = 0.87 [0.72 to 1.05], *p* = 0.153). When the benzodiazepines were compared with the z-drugs (Table H in S1 Appendix), there were no significant differences for out-of-hospital or total mortality.

**Table 2:** Unadjusted incidence of death (per 1,000 person-years) during follow-up according to study hypnotic and concurrent opioid use.

	Benzodiazepines		z-drugs		Trazodone	
*No concurrent opioid use*						
Person-years	32,388		26,497		16,177	
	*Deaths*	*Rate (95% CI)*	*Deaths*	*Rate (95% CI)*	*Deaths*	*Rate (95% CI)*
Out of hospital	260	8.0 (7.1 to 9.0)	150	5.7 (4.8 to 6.6)	156	9.6 (8.1 to 11.2)
In hospital	158	4.9 (4.1 to 5.6)	77	2.9 (2.3 to 3.6)	100	6.2 (5.0 to 7.4)
All	418	12.9 (11.7 to 14.1)	227	8.6 (7.5 to 9.7)	256	15.8 (13.9 to 17.8)
*Concurrent opioid use*						
Person-years	4,278		3,541		2,347	
	*Deaths*	*Rate (95% CI)*	*Deaths*	*Rate (95% CI)*	*Deaths*	*Rate (95% CI)*
Out of hospital	90	21.0 (16.7 to 25.4)	40	11.3 (7.8 to 14.8)	19	8.1 (4.5 to 11.7)
In hospital	37	8.6 (5.9 to 11.4)	24	6.8 (4.1 to 9.5)	17	7.2 (3.8 to 10.7)
All	127	29.7 (24.5 to 34.8)	64	18.1 (13.6 to 22.5)	36	15.3 (10.3 to 20.3)

#### Concurrent opioid

During 10,166 person-years of follow-up with concurrent opioid use, there were 4,278 person-years and 90 out-of-hospital deaths for benzodiazepines (21.0/1,000), 3,541 person-years and 40 deaths for z-drugs (11.3/1,000), and 2,347 person-years and 19 deaths (8.1/1,000) for trazodone (Table 2). When compared with trazodone (Fig 2), the risk of out-of-hospital death was increased for both benzodiazepines (HR = 3.02 [1.83 to 4.97], *p* < 0.001) and z-drugs (HR = 1.98 [1.14 to 3.44], *p* = 0.015). The increased risk remained present when the out-of-hospital deaths were restricted to those with cardiovascular causes (Table 3). The adjusted risk of total mortality for both benzodiazepines (HR = 2.21 [1.52 to 3.20], *p* < 0.001) and z-drugs (HR = 1.65 [1.09 to 2.50], *p* = 0.018) was greater than that for trazodone. When the benzodiazepines were compared with the z-drugs (Table H in S1 Appendix), there were no significant differences for out-of-hospital or total mortality.

**Table 3:** Sensitivity analyses for benzodiazepines and z-drugs vs. trazodone, according to concurrent opioid use.

	No concurrent opioid				Concurrent opioid			
	Benzodiazepines		z-drugs		Benzodiazepines		z-drugs	
	HR (95% CI)	*p*-value	HR (95% CI)	*p*-value	HR (95% CI)	*p*-value	HR (95% CI)	*p*-value
*Primary analysis*								
Out-of-hospital deaths	0.99 (0.81 to 1.22)	0.954	0.96 (0.76 to 1.23)	0.767	3.02 (1.83 to 4.97)	<0.001	1.98 (1.14 to 3.44)	0.015
All deaths	0.95 (0.80 to 1.12)	0.513	0.87 (0.72 to 1.05)	0.153	2.21 (1.52 to 3.20)	<0.001	1.65 (1.09 to 2.49)	0.018
**(a) Subgroups of interest**								
*Overdose deaths excluded*								
Out-of-hospital deaths	1.00 (0.81 to 1.23)	0.977	0.96 (0.75 to 1.22)	0.738	2.98 (1.81 to 4.91)	<0.001	1.99 (1.14 to 3.45)	0.015
All deaths	0.95 (0.81 to 1.12)	0.527	0.87 (0.72 to 1.05)	0.144	2.19 (1.51 to 3.18)	<0.001	1.65 (1.09 to 2.49)	0.017
*Cardiovascular deaths only*								
Out-of-hospital deaths	0.99 (0.77 to 1.28)	0.948	0.94 (0.70 to 1.26)	0.660	3.41 (1.79 to 6.51)	<0.001	2.65 (1.32 to 5.30)	0.006
All deaths	0.92 (0.74 to 1.14)	0.453	0.83 (0.65 to 1.07)	0.153	2.24 (1.38 to 3.63)	0.0012	1.68 (0.98 to 2.88)	0.059
*Benzodiazepines hypnotic label or sleep problem diagnosis*								
Out-of-hospital deaths	0.85 (0.66 to 1.10)	0.223			2.67 (1.54 to 4.64)	<0.001		
All deaths	0.84 (0.69 to 1.03)	0.089			1.85 (1.20 to 2.85)	0.0050		
**(b) Alternative statistical analysis**^**a**^								
*Clustering by region*: *variance adjustment*								
Out-of-hospital deaths	0.99 (0.80 to 1.24)	0.960	0.96 (0.75 to 1.23)	0.740	3.02 (1.83 to 4.99)	<0.001	1.99 (1.14 to 3.46)	0.015
All deaths	0.95 (0.80 to 1.12)	0.529	0.87 (0.71 to 1.06)	0.157	2.21 (1.52 to 3.21)	<0.001	1.65 (1.09 to 2.50)	0.018
*Cluster by region*: *control for and variance adjustment*								
Out-of-hospital deaths	0.99 (0.80 to 1.24)	0.959	0.96 (0.75 to 1.23)	0.740	3.02 (1.83 to 4.99)	<0.001	1.99 (1.14 to 3.46)	0.015
All deaths	0.95 (0.80 to 1.12)	0.528	0.87 (0.71 to 1.06)	0.158	2.21 (1.52 to 3.21)	<0.001	1.65 (1.09 to 2.50)	0.018
*Covariates fixed at baseline*								
Out-of-hospital deaths	0.93 (0.76 to 1.15)	0.520	0.94 (0.73 to 1.20)	0.590	2.81 (1.71 to 4.63)	<0.001	1.97 (1.14 to 3.43)	0.016
All deaths	0.93 (0.79 to 1.09)	0.377	0.86 (0.70 to 1.04)	0.118	2.14 (1.47 to 3.11)	<0.001	1.65 (1.09 to 2.49)	0.018
*Time-dependent propensity score weights*								
Out-of-hospital deaths	1.09 (0.88 to 1.35)	0.407	1.03 (0.80 to 1.32)	0.822	2.80 (1.67 to 4.71)	<0.001	2.13 (1.18 to 3.82)	0.013
All deaths	1.03 (0.87 to 1.22)	0.113	0.93 (0.76 to 1.13)	0.478	2.03 (1.38 to 3.00)	<0.001	1.63 (1.06 to 2.51)	0.026
*Propensity score matched*, *covariates fixed at baseline*								
Out-of-hospital deaths	0.95 (0.74 to 1.21)	0.674	1.04 (0.78 to 1.39)	0.800	2.39 (1.37 to 4.16)	0.002	2.00 (1.01 to 3.95)	0.046
All deaths	0.97 (0.80 to 1.18)	0.732	0.99 (0.79 to 1.24)	0.919	2.05 (1.33 to 3.18)	0.001	1.73 (1.07 to 2.78)	0.025

HRs adjusted for study covariates.

^a^See Section I in S1 Appendix for details of analysis.

HR, hazard ratio.

### Sensitivity analyses

HR estimates were little changed with the exclusion of drug overdose deaths, restriction of deaths to those with cardiovascular causes, or limiting benzodiazepines to patients who started a drug with a hypnotic label or had an insomnia diagnosis prior to cohort entry (Table 3). The HRs estimated with alternative statistical methods did not differ materially from those of the primary analysis.

## Discussion

In the absence of concurrent opioids, US Medicare beneficiaries 65 years of age or older without severe illness who began use of either benzodiazepine hypnotics or z-drugs had out-of-hospital and total mortality that differed little from that of comparable patients starting trazodone. In contrast, with concurrent opioids, patients receiving benzodiazepines or z-drugs had significantly increased out-of-hospital and total mortality relative to trazodone. The association with increased risk persisted with the exclusion of overdose deaths. There were no significant differences in mortality between patients receiving benzodiazepines and z-drugs, either without or with concurrent opioids.

Our finding in patients without opioid use of no significantly increased mortality for benzodiazepines or z-drugs must be interpreted cautiously. To reduce confounding by both the adverse effects of inadequate sleep [6,10] and the association of hypnotic use with life-threatening illness [45], the study comparator was a hypnotic from a different pharmacologic class. Consequently, our findings could be influenced by the cardiovascular and other adverse effects of trazodone [26,27,56]. Indeed, a 2017 clinical guideline discouraged the use of trazodone as a hypnotic [56] given the paucity of clinical trial data supporting its efficacy and safety. However, both patients and providers consider trazodone a safer alternative to benzodiazepine and z-drug hypnotics [56], and trazodone prescribing for insomnia is increasing [5]. Further studies of the efficacy and safety of trazodone for insomnia are needed.

At baseline, 12.5% of cohort members initiating treatment with benzodiazepine hypnotics had concurrent opioids, which is consistent with other reports from older populations [29–31]. Our data indicate that the hazards of concurrent use go beyond the documented association of benzodiazepines with increased opioid overdose deaths [57], which are relatively uncommon in older populations [38]. The greater than 2-fold increase in total mortality we observed, even after overdose deaths were excluded, suggests that concurrent benzodiazepine–opioid exposure poses a major risk to the health of older persons.

Although the z-drugs were introduced as a safer alternative to the benzodiazepine hypnotics [7], more recent thinking is that the adverse effects of these 2 hypnotic classes are similar [10]. However, although there is evidence of z-drug involvement in opioid overdose [58–60], unlike benzodiazepines [28], there is no “black box” FDA warning against opioid coadministration. Our findings of increased out-of-hospital and total mortality suggest that in combination with opioids, the z-drugs may be more hazardous than previously thought.

Because both hypnotics and opioids may be prescribed for patients with greater likelihood of death, a key element of our design was an active control: comparing benzodiazepines or z-drug exposure, with or without concurrent opioids, to comparable use of low-dose trazodone. Furthermore, the time-dependent propensity score analysis controlled for a large number of possible confounders. The distribution of baseline covariates in patients with concurrent opioid use indicated that patients receiving trazodone had greater comorbidity than did users of the other hypnotics, particularly relative to the z-drugs. Consequently, if there is residual confounding, our findings could underestimate the risks of coadministration of opioids with benzodiazepine hypnotics or z-drugs.

We postulated that the adverse cardiovascular effects of nocturnal respiratory impairment could mediate an increased risk of death for the benzodiazepines and z-drugs. However, our primary endpoint included all out-of-hospital deaths, both to avoid potentially differential under-ascertainment of cardiovascular deaths [37,46–48] and to include deaths related to other adverse respiratory effects of study hypnotics and opioids [22,36]. Although the increased risk for out-of-hospital deaths from cardiovascular causes is consistent with our hypothesis, the study data did not permit elaboration of the mechanisms of hypnotic-related deaths.

The most recent study data were for calendar 2015. Since that time, efforts to reduce opioid use have intensified, including the FDA black box warning against concurrent use with benzodiazepines [28]. Nevertheless, more recent data demonstrate continuing elevated prevalence of concurrent use of benzodiazepines and z-drugs with opioids [30,31].

The study cohort consisted of US Medicare fee-for-service beneficiaries 65 years of age or older without severe illnesses, which limits generalizability. Prescribing patterns for both hypnotics and opioids may differ in other countries. Findings may differ for younger patients (although those over 65 years of age have greater prevalence of hypnotic use [1,2,61] and increased mortality rates), those in Medicare managed care, or with substance use disorder.

There were several other study limitations. For patients receiving z-drugs with concurrent opioids, the total number of deaths was small, and the lower bound of the HR 95% CI was 1.09. Thus, a relatively small additional risk for this exposure cannot be ruled out. Although all of the study drugs are widely prescribed as hypnotics and included in guidelines for insomnia treatment [6], some benzodiazepines have other indications. However, findings did not change with exclusion of patients receiving benzodiazepines for whom the indication was most likely to be ambiguous. Study benzodiazepines only included those with a hypnotic indication or mentioned in insomnia treatment guidelines, even though other benzodiazepines are prescribed as hypnotics in clinical practice. There was no comparison to patients whose insomnia was treated with less frequently prescribed hypnotic medications or cognitive behavioral therapy. We did not examine differences between the study hypnotics for many potentially important nonfatal endpoints. Finally, the study Medicare data did not include physiological data that could be useful for identifying patients at greatest risk of hypnotic–opioid adverse effects.

## Conclusions

In US Medicare beneficiaries 65 years of age or older without concurrent opioids who initiated treatment with benzodiazepine hypnotics, z-drugs, or low-dose trazodone, study hypnotics were not associated with mortality. With concurrent opioids, benzodiazepines and z-drugs were associated with increased out-of-hospital and total mortality. These findings indicate that the dangers of benzodiazepine–opioid coadministration go beyond the documented association with overdose death and suggest that in combination with opioids, the z-drugs may be more hazardous than previously thought.

## Supporting information

S1 Checklist STROBE guideline. STROBE, Strengthening the Reporting of Observational Studies in Epidemiology.
(DOCX)

S1 Protocol The prospective plan for study design and analysis. (DOCX)

S1 Appendix Details of study methodology and additional results (Sections A–K, Tables A–H, and Fig A).
(PDF)

## Abbreviations

CMS: Center for Medicare & Medicaid Services
FDA: US Food and Drug Administration
GABA-A: γ-aminobutyric acid type A
HR: hazard ratio
MME: morphine milligram equivalents
NDI: National Death Index
STROBE: Strengthening the Reporting of Observational Studies in Epidemiology
VRDC: Virtual Research Data Center
