# Supplementary material for: Mortality and concurrent use of opioids and hypnotics in older patients: A retrospective cohort study
Source: PLoS Med. 2021 Jul 15;18(7):e1003709. doi: 10.1371/journal.pmed.1003709 (PMC8321368; doi:10.1371/journal.pmed.1003709)
Supplement: S1 Protocol — (DOCX) [file pmed.1003709.s002.docx]

**S1 Protocol**

**Extract from Specific Aims (NIH grant submission, grant funded, National Heart, Lung, and Blood Institute [HL081707])**

Hypnotic prescriptions for U.S. adults increased from 5.3 million in 1999 to 20.8 million in 2010, with the most frequent use by persons 65 years of age or older.^1,2^ The 3 most commonly prescribed hypnotics are the benzodiazepines, selective benzodiazepine receptor agonists (sBzRAs), and trazodone.^3^ Observational studies of hypnotics and mortalityconsistently have found that benzodiazepines and sBzRAs are associated with a 60 to 73% increased risk of all-cause mortality.^4,5^ Controversial biological plausibility and study limitations have led most to conclude that the association is not causal.^6,7^

One mechanism by which benzodiazepines and sBzRAs could increase mortality is nocturnal respiratory impairment, which causes apnea, hypoxemia and hypercapnia. These are associated with sympathetic activation and increased risk of cardiac arrhythmias and cardiovascular death,^8-12^ the most common cause of death in the U.S.^13^ Benzodiazepines impair respiration: they decrease respiratory muscle strength and endurance,^14^ decrease oxyhemoglobin saturation (SpO_2_) in anesthetized patients,^15^ produce obstructive apnea and hypopnea in overdose,^16^ impair minute ventilation, decrease SpO_2_ and cause hypercapnia in patients with chronic obstructive pulmonary disease,^17,18^ have synergistic respiratory depressant effects in combination with opioids^19,20^ that increase the risk of fatal overdoses,^21^ and reduce minimum nocturnal SpO_2_ in patients with sleep apnea.^22^ The hypothesis that these adverse respiratory effects increase cardiovascular risk is supported by our preliminary data showing a dose-related increase in the incidence of out-of-hospital cardiovascular deaths (most commonly sudden cardiac death) for benzodiazepines (§a.2.d). The sBzRAs impair respiration to a lesser degree^22-27^ and the available data indicate trazodone causes minimal, if any, respiratory impairment.^27-32^ Thus, trazodone could have better cardiovascular safety than the benzodiazepines and possibly the sBzRAs.

Opioid analgesics could potentiate the adverse cardiovascular effects of benzodiazepines and sBzRAs. Concurrent opioid-hypnotic use is frequent; in 2014, an estimated 26% of patients with a benzodiazepine prescription also had an opioid prescription.^33^ Opioid analgesics cause a dose-related impairment in all phases of respiratory activity^34,35^ and cause or exacerbate sleep-disordered breathing.^34,36,37^ We recently found^38^ that long-acting opioid users had a 65% increase in the risk of out-of-hospital cardiovascular death (hazard ratio = 1.65 [1.10-2.46]), evidence that medications that impair nocturnal respiration have adverse cardiovascular effects. Combined respiratory effects of benzodiazepines and opioids also could increase cardiovascular risk, a hypothesis supported by our preliminary data indicating a synergistic increase in cardiovascular death risk with concurrent benzodiazepine-long-acting opioid use. Thus, the adverse cardiovascular effects of some hypnotics might only be present in combination with opioids.

Despite the use of prescribed hypnotics by millions of patients, the potential association with increased mortality has not materially affected clinical practice or public health policy because it is nearly universally considered as due to bias. However, an important harm of benzodiazepines and sBzRAs may have been overlooked. The nocturnal respiratory impairment-cardiovascular death mechanism provides a biologically plausible basis for this association and suggests clinically meaningful between-drug differences. Some limitations of extant studies could have underestimated the true risk. Thus, rigorous study of the relative cardiovascular safety of frequently prescribed hypnotics, without and with concurrent opioids, is needed to guide clinical practice.

We plan to study the cardiovascular effects of hypnotics in Medicare enrollees—who have the most frequent hypnotic use and greatest susceptibility to increased mortality—to test the hypotheses that:

**Aim 1. In the absence of opioid use, the risk of out-of-hospital cardiovascular and total mortality for benzodiazepine and sBzRA users is greater than that for trazodone users.**

**Aim 2. Concurrent use of opioid analgesics potentiates the risk of cardiovascular mortality for patients with benzodiazepine or sBzRA use.**

**Extract from Analysis Plan (HL081707)**

1. Sources of Data

We will obtain study data from computerized files of medical encounters for Medicare beneficiaries maintained by the CMS Research Data Assistance Center (ResDAC). We will restrict our request to beneficiaries with medication coverage (enrolled part D) in a fee-for-service plan (not enrolled part C, Medicare Advantage) and not enrolled because of disability.

2. Study Hypnotics

All prescriptions for drugs with an FDA-approved hypnotic indication are considered hypnotics because off-label use is minimal, given the rapid onset of drowsiness. Other drugs frequently prescribed for insomnia that guidelines describe as hypnotics^6,39^ are considered hypnotics if the dosing schedule is once/day and there is no evidence of a different indication.

3. Cohort Inclusion/Exclusion

Cohort eligibility criteria are designed to identify patients beginning a course of hypnotic therapy, with no past use of any benzodiazepine or trazodone, with adequate information in the Medicare files to determine study covariates and who are unlikely to have a short-term elevated risk of death from a known condition. As in our previous studies,^38^ we exclude patients with serious illness that poses short-term increased risk of death to reduce confounding by hypnotic use in terminally ill patients and to decrease the likelihood that cardiovascular deaths are related to prior illness. We do not require a sleep disorder diagnosis because this diagnosis often is not recorded in clinical practice.

4. Covariates

A key design element is controlling for potential confounders that vary according to the three types of hypnotics. Thus, as in our long-acting opioid study (§a.3.b), we will tightly match the groups according to covariates with a plausible direct or indirect relation to both hypnotic initiation and cardiovascular or total mortality; as in our previous studies^38^ there will be more than 100 covariates.

5. Endpoints

Out-of-hospital cardiovascular death is an appropriate clinical endpoint to study medication-related respiratory impairment in large populations because 1) it can be efficiently ascertained from death certificates and,2) for patients with no life-threatening illness, most such deaths are sudden cardiac deaths. Out-of-hospital cardiovascular deaths will be identified from the death certificate underlying cause of death based on the ACC/AHA criteria.^40^

Total mortality is a coprimary endpoint for two reasons. First, some hypnotics plausibly could increase the risk of death from non-cardiovascular causes; for example, both benzodiazepines and sBzRAs are linked to increased risk of injuries.^41-44^ Second, this endpoint guards against differential misclassification of cause of death; for example, a nocturnal death might be more frequently classified as an overdose for benzodiazepine patients than for trazodone patients.

6. Analysis

The analysis will include three *a priori* comparisons: benzodiazepine vs trazodone, sBzRA vs trazodone, and benzodiazepine vs sBzRA for each of the two coprimary endpoints. We will not adjust for multiple comparisons, given that each comparison is planned, *a priori* and hypothesis-based; however, p-values will be calculated permitting Bonferroni corrections. Relative risk will be estimated with HRs calculated from Cox regression models.

**Material changes from Original Analysis Plan**

1. As more accurate sample size estimation became possible, we realized that the propensity-score matching originally envisioned would materially reduce sample size and power. Thus, the primary analysis used propensity-score stratification. A sensitivity analysis was performed with propensity-score matching.

2. We changed the primary endpoint to include all out-of-hospital deaths, the primary endpoint for our previous investigation of opioids in chronic non-cancer pain.^45^ We made this change for two reasons. First, we became concerned that out-of-hospital cardiovascular deaths would be under-ascertained. Second, the broader endpoint captures the respiratory-related deaths that occasionally are a consequence of some study hypnotics and opioids.

Reference List

1. Ford ES, Wheaton AG, Cunningham TJ, Giles WH, Chapman DP, Croft JB. Trends in outpatient visits for insomnia, sleep apnea, and prescriptions for sleep medications among US adults: findings from the National Ambulatory Medical Care survey 1999-2010. *Sleep.* 2014;37(8):1283-1293.

2. Chong Y, Fryer CD, Gu Q. Prescription sleep aid use among adults: United States, 2005-2010. *NCHS Data Brief.* 2013(127):1-8.

3. Bertisch SM, Herzig SJ, Winkelman JW, Buettner C. National use of prescription medications for insomnia: NHANES 1999-2010. *Sleep.* 2014;37(2):343-349.

4. Kripke DF. Mortality risk of hypnotics: strengths and limits of evidence. *Drug Saf.* 2016;39(2):93-107.

5. Parsaik AK, Mascarenhas SS, Khosh-Chashm D, et al. Mortality associated with anxiolytic and hypnotic drugs-A systematic review and meta-analysis. *Aust N Z J Psychiatry.* 2015.

6. Buysse DJ. Insomnia. *JAMA.* 2013;309(7):706-716.

7. Neutel CI, Johansen HL. Association between hypnotics use and increased mortality: causation or confounding? *Eur J Clin Pharmacol.* 2015;71(5):637-642.

8. Mehra R, Redline S. Arrhythmia risk associated with sleep disordered breathing in chronic heart failure. *Curr Heart Fail Rep.* 2014;11(1):88-97.

9. Mansukhani MP, Wang S, Somers VK. Sleep, death, and the heart. *Am J Physiol Heart Circ Physiol.* 2015;309(5):H739-H749.

10. Monahan K, Storfer-Isser A, Mehra R, et al. Triggering of nocturnal arrhythmias by sleep-disordered breathing events. *J Am Coll Cardiol.* 2009;54(19):1797-1804.

11. Gami AS, Olson EJ, Shen WK, et al. Obstructive sleep apnea and the risk of sudden cardiac death: a longitudinal study of 10,701 adults. *J Am Coll Cardiol.* 2013;62(7):610-616.

12. Mehra R, Benjamin EJ, Shahar E, et al. Association of nocturnal arrhythmias with sleep-disordered breathing. *Am J Respir Crit Care Med.* 2006;173:910-916.

13. Jiaquan Xu M, Sherry L.Murphy BS, Kenneth D.Kochanek MA, and Brigham A.Bastian BS, Statistics DoV. Deaths: Final Data for 2013. *National Vital Statistics Reports.* 2016;64(2).

14. Sanger DJ, Benavides J, Perrault G, et al. Recent developments in the behavioral pharmacology of benzodiazepine (omega) receptors: evidence for the functional significance of receptor subtypes. *Neurosci Biobehav Rev.* 1994;18(3):355-372.

15. Quario RL, Thompson C. Efficacy of propofol compared to midazolam as an intravenous premedication agent. *Minerva Anestesiol.* 2008;74(5):173-179.

16. Gueye PN, Lofaso F, Borron SW, et al. Mechanism of respiratory insufficiency in pure or mixed drug-induced coma involving benzodiazepines. *J Toxicol Clin Toxicol.* 2002;40(1):35-47.

17. Battaglia S, Bezzi M, Sferrazza Papa GF. Are benzodiazepines and opioids really safe in patients with severe COPD? *Minerva Med.* 2015.

18. Vozoris NT, Fischer HD, Wang X, et al. Benzodiazepine drug use and adverse respiratory outcomes among older adults with COPD. *Eur Respir J.* 2014;44(2):332-340.

19. White JM, Irvine RJ. Mechanisms of fatal opioid overdose. *Addiction.* 1999;94(7):961-972.

20. Jones CM, McAninch JK. Emergency department visits and overdose deaths from combined use of opioids and benzodiazepines. *Am J Prev Med.* 2015;49(4):493-501.

21. Park TW, Saitz R, Ganoczy D, Ilgen MA, Bohnert AS. Benzodiazepine prescribing patterns and deaths from drug overdose among US veterans receiving opioid analgesics: case-cohort study. *BMJ.* 2015;350:h2698.

22. Mason M, Cates CJ, Smith I. Effects of opioid, hypnotic and sedating medications on sleep-disordered breathing in adults with obstructive sleep apnoea. *Cochrane Database Syst Rev.* 2015;7:CD011090.

23. Elliot EE, White JM. The acute effects of zolpidem compared to diazepam and lorazepam using radiotelemetry. *Neuropharmacology.* 2001;40(5):717-721.

24. Gunja N. The clinical and forensic toxicology of Z-drugs. *J Med Toxicol.* 2013;9(2):155-162.

25. Zhang XJ, Li QY, Wang Y, Xu HJ, Lin YN. The effect of non-benzodiazepine hypnotics on sleep quality and severity in patients with OSA: a meta-analysis. *Sleep Breath.* 2014;18(4):781-789.

26. Girault C, Muir JF, Mihaltan F, et al. Effects of repeated administration of zolpidem on sleep, diurnal and nocturnal respiratory function, vigilance, and physical performance in patients with COPD. *Chest.* 1996;110(5):1203-1211.

27. Roth T. Hypnotic use for insomnia management in chronic obstructive pulmonary disease. *Sleep Med.* 2009;10(1):19-25.

28. Bossini L, Casolaro I, Koukouna D, Cecchini F, Fagiolini A. Off-label uses of trazodone: a review. *Expert Opin Pharmacother.* 2012;13(12):1707-1717.

29. Fagiolini A, Comandini A, Catena DOM, Kasper S. Rediscovering trazodone for the treatment of major depressive disorder. *CNS Drugs.* 2012;26(12):1033-1049.

30. Generali JA, Cada DJ. Trazodone: Insomnia (Adults). *Hosp Pharm.* 2015;50(5):367-369.

31. Eckert DJ, Malhotra A, Wellman A, White DP. Trazodone increases the respiratory arousal threshold in patients with obstructive sleep apnea and a low arousal threshold. *Sleep.* 2014;37(4):811-819.

32. Smales ET, Edwards BA, Deyoung PN, et al. Trazodone effects on obstructive sleep apnea and non-REM arousal threshold. *Ann Am Thorac Soc.* 2015;12(5):758-764.

33. Hwang CS, Kang EM, Kornegay CJ, Staffa JA, Jones CM, McAninch JK. Trends in the concomitant prescribing of opioids and benzodiazepines, 2002-2014. *Am J Prev Med.* 2016.

34. Zutler M, Holty JE. Opioids, sleep, and sleep-disordered breathing. *Curr Pharm Des.* 2011;17(15):1443-1449.

35. Boyer EW. Management of opioid analgesic overdose. *N Engl J Med.* 2012;367:146-155.

36. Yue HJ, Guilleminault C. Opioid medication and sleep-disordered breathing. *Med Clin North Am.* 2010;94(3):435-446.

37. Lee-Iannotti J, Parish JM. The epidemic of opioid use: implications for the sleep physician. *J Clin Sleep Med.* 2014;10(6):645-646.

38. Ray WA, Chung CP, Murray KT, Hall K, Stein CM. Prescription of long-acting opioids and mortality in patients with chronic noncancer pain. *JAMA.* 2016;315:2415-2423.

39. Winkelman JW. Clinical practice: Insomnia disorder. *N Engl J Med.* 2015;373(15):1437-1444.

40. Hicks KA, Tcheng JE, Bozkurt B, et al. 2014 ACC/AHA key data elements and definitins for cardiovascular endpoint events in clinical trials. *JACC.* 2015;66(4):403-469.

41. Ray WA, Griffin MR, Downey W. Benzodiazepines of long and short elimination half-life and the risk of hip fracture. *JAMA.* 1989;262:3303-3307.

42. Ray WA, Thapa P, Gideon P. Benzodiazepines and the risk of falls in nursing home residents. *J Am Geriatr Soc.* 2000;48:682-685.

43. Finkle WD, Der JS, Greenland S, et al. Risk of fractures requiring hospitalization after an initial prescription for zolpidem, alprazolam, lorazepam, or diazepam in older adults. *J Am Geriatr Soc.* 2011;59(10):1883-1890.

44. Hansen RN, Boudreau DM, Ebel BE, Grossman DC, Sullivan SD. Sedative hypnotic medication use and the risk of motor vehicle crash. *Am J Public Health.* 2015;105(8):e64-e69.

45. Ray WA, Chung CP, Murray KT, Cooper WO, Hall K, Stein CM. Out-of-hospital mortality among patients receiving methadone for noncancer pain. *JAMA Intern Med.* 2015;175(3):420-427.
