# Supplementary material for: Mortality and concurrent use of opioids and hypnotics in older patients: A retrospective cohort study
Source: PLoS Med. 2021 Jul 15;18(7):e1003709. doi: 10.1371/journal.pmed.1003709 (PMC8321368; doi:10.1371/journal.pmed.1003709)
Supplement: S1 Appendix — Details of study methodology and additional results (Sections A–K, Tables A–H, and Fig A). (PDF) [file pmed.1003709.s003.pdf]

Appendix

This appendix provides additional details for the study of prescription hypnotics and should be read in conjunction with the primary manuscript (MS).

A. Study Medications: Prescription Hypnotics

The study hypnotics (Table A) include all prescriptions for benzodiazepines with an FDA-approved hypnotic indication or z-drugs. They also include other benzodiazepines--alprazolam, clonazepam and lorazepam--frequently prescribed for insomnia and included in hypnotic guidelines.[1,2] Trazodone was considered a hypnotic if the dose did not exceed 100mg, often recommended for hypnotic use. Although higher doses may be prescribed for insomnia, these potentially indicate treatment of depression.[3]

For all study medications, the prescribed regimen had to be consistent with hypnotic use: a single tablet/capsule per day with allowance for tablets that can be divided to provide a lower daily dose (e.g., trazodone 25mg). We also excluded patients with a diagnosis in the past 90 days of an alternative indication for either benzodiazepines or trazodone: panic disorder, anxiety disorder/post-traumatic stress disorder, somnambulism, parasomnia, periodic limb movement disorder, myoclonus, restless legs, spasticity, epilepsy/recurrent seizures/convulsions, major depression, bipolar disorder, or other mood disorder.

Table A. Study hypnotics with standard doses.

Drug	Dose, standard, mg[a]
A. Benzodiazepines	
Alprazolam	0.25
Clonazepam	0.5
Estazolam	1
Flurazepam	15
Lorazepam	.25
Quazepam	7.5
Temazepam	15
Triazolam	.125
B. z-Drugs	
Eszopiclone	1
Zaleplon	5
Zolpidem, oral tablet	5
Zolpidem, ER	6.25
Zolpidem, sublingual, Intermezzo	1.75
Zolpidem, sublingual, Edulan	5
Zolpidem, oral spray	5
C. Trazodone	
Trazodone, ≤100mg	25

[a]Low end of usual hypnotic dose range in adults. Source for all except alprazolam: Buysse DJ. Insomnia. *JAMA.* 2013;309(7):706-716. Source for alprazolam: Strand MC, Morland J, Slordal L, et al. Conversion factors for assessment of driving impairment after exposure to multiple benzodiazepines/z-hypnotics or opioids. *Forensic Sci Int.* 2017;281:29-36.

The study was restricted to hypnotics with oral or sublingual route, thus excluding IV or injectable drugs. It also excluded medications given in hospitals, physician offices or clinics. The study medications did not include older hypnotics (e.g., barbiturates) or hypnotics introduced more recently (ramelteon, suvorexant, tasimelteon) because use was infrequent during the study period nor did they include other medications with limited hypnotic use (doxepin, amitriptyline, gabapentin, olanzapine, quetiapine, risperidone).

Use of non-study benzodiazepines in the past year disqualified patients for cohort entry. These included clorazepate, chlordiazepoxide, chlordiazepoxide-amitriptyline, diazepam, and oxazepam, but did not include the amnestic midazolam.

We defined current hypnotic use as the day of the prescription fill through the end of the days of supply, offset by one day because hypnotics generally are taken in the evening and thus hypnotic-related deaths should occur either during the night or on the next day. If the patient entered the hospital, current use status was frozen for the hospital stay. If the hospital stay was no more than 7 days, we extended the days of supply by the length of stay to account for the patient receiving the medication in the hospital. For longer hospital stays, we assumed there was a greater likelihood that the prescription would be changed in the hospital and thus set the status after discharge to noncurrent use, unless a prescription was filled on discharge.

B. Study Medications: Opioids

The study opioids (Table B) excluded parenteral opioids (infrequently prescribed for outpatients) and preparations specifically formulated for cough or diarrhea. Although methadone is prescribed as opioid

replacement therapy, it also is frequently used for pain and thus was included in the study opioids. Although patients with baseline use of buprenorphine were excluded, use during followup was not. Opioids were classified as short- or long-acting and dose-equivalents were calculated in morphine-milligram equivalents (MME) according to guidelines for chronic opioid therapy for non-cancer pain (Table B).[4]

Appendix Table B. Study opioid analgesics, with MME conversion factors[a]. These do not include the antitussive levopropoxyphene, cough preparations, tincture of opium for diarrhea, and hydrocodone in cough preparations, both tablet (e.g., Hycodan) and syrup/liquid.

Drug	Conversion Factor	Standard dose
A. Short-Acting Opioid Analgesics		
Butorphanol, intranasal	7	4.3
Codeine	0.15	200
Dihydrocodeine	0.25	120
Fentanyl, buccal/SL tablet or lozenge/troche (mcg)	.13	240 mcg
Fentanyl, film or oral spray	.18	167 mcg
Fentanyl, nasal spray	.16	188 mcg
Hydrocodone	1.0	30
Hydromorphone	4.0	7.5
Meperidine	0.1	300
Morphine, not SR	1.0	30
Oxycodone, not SR	1.5	20
Oxymorphone	3.0	10
Pentazocine	0.37	81
Propoxyphene HCL	0.23	130
Propoxyphene napsylate	0.15	200
Tapentadol	0.23	133
Tramadol	0.4	75
B. Long-Acting Opioid Analgesics		
Buprenorphine, buccal film (mcg)	.03	1000mcg
Buprenorphine, tablet or film	30	1
Buprenorphine, transdermal patch (mcg/hr)	1.8	22.33 mcg/hr
Fentanyl, transdermal patch(mcg/hr)	2.4	12.5 mcg/hr
Hydrocodone ER	1.0	30
Hydromorphone, ER	4.0	7.5
Levorphanol	11.0	2.7
Methadone	3.0	10
Morphine, SR/ER	1.0	30
Oxycodone, SR	1.5	20
Oxymorphone, ER	3.0	10
Tapentadol, ER	0.23	133
Tramadol ER/VR	0.1	300

[a]National Center for Injury Prevention and Control. CDC compilation of benzodiazepines, muscle relaxants, stimulants, zolpidem, and opioid analgesics with oral morphine milligram equivalent conversion factors, 2018 version. Atlanta, GA: Centers for Disease Control and Prevention; 2018. Available at https:// www.cdc.gov/drugoverdose/resources/data.html.

C. Cohort

C.1 Data Availability

The study Medicare data reside in the Center for Medicare & Medicaid Services (CMS) Chronic Condition Warehouse. The data may be obtained by applying to the Research Data Assistance Center (RESDAC) at the University of Minnesota in the U.S. The process begins with contact to RESDAC, either by visiting the website (https://resdac.org/), phone (1-888-973-7322), or email (resdac@umn.edu). RESDAC has detailed descriptions of the data and procedures for obtaining access, with staff available to assist researchers.

C.2 Cohort Eligibility Criteria

Appendix Table C. Cohort eligibility criteria

Criterion	N
**Hypnotic prescription identified by Chronic Condition Warehouse.** Filled Part D prescription for study hypnotic between 1/1/2014 and 29 September 2015 with beneficiary 65 years of age or older on the fill date and during the month of the fill date enrolled in Medicare parts A, B, and D but not in part C. The fill day is t_0-1 and the day following the fill date is t_0.	4,819,081
**Age.** Age 65 years of age or older on t_0.	4,802,272
**Enrollment.** Date of birth and sex known and enrolled in Medicare parts A, B, and D in the preceding 12 months, with no Part C enrollment in any of those 12 months. Excludes Medicare Advantage beneficiaries because recording of medical care encounters may be less complete.	4,231,166
**Hypnotic regimen.** Prescription consistent with a hypnotic regimen of 2 or more days with either one-half or one dispensed unit per day of supply.	2,886,289
**New user.** For the period [t_0-365, t_0-1][a], no other filled prescription for any drug that potentially is a hypnotic, with the exception of a single prescription with only 1 day of supply (periprocedural). This thus excludes patients with past use of:<br>a. Study hypnotics (including trazodone in any dose) or non-study benzodiazepines**;**<br>b. Older hypnotics (amobarbital, butabarbital, chloral hydrate, ethchlorvynol, secobarbital) or newer hypnotics (ramelteon, suvorexant, tasimelteon)<br>c. Medications with use of medications occasionally prescribed for insomnia (doxepin, amitriptyline, gabapentin, olanzapine, quetiapine, risperidone) with an insomnia diagnosis on the day or within 7 days of the prescription fill.	841,625
**Alternative indication.** No evidence in [t_0-90,t_0-1] of an alternative indication (§1).	655,758
**Medical history.** At least one outpatient visit (including ED, excluding lab) and one filled prescription in [t_0 -365, t_0-2], to assure regular contact with medical care prior to cohort entry.	646,226
**Long-term care.** Not residing in a nursing home or other residential institution in the interval [t_0-365, t_0-1], except for stays of <30 days following hospital discharge. This includes inferred nursing home stays, defined as 2 or more outpatient encounters in the interval [t_0-365, t_0-1] with procedure indicating nursing home place of service separated by at least 28 days. Also includes external cause of injury diagnosis code (E849.7) indicating institution residence.	578,095
**Hospice.** No evidence of hospice stay in [t_0-365, t_0-1].	573,825
**Hospital stays[b] indicating unstable health.** Excludes a) those in hospital past 30 days ([t_0-30, t_0-1]) for whom both deaths and hypnotic start potentially related to hospital admission; b) more than 30 days of total hospital stay in past year ([t_0-365, t_0-1]); or c) more than two hospital admissions in past year ([t_0-365, t_0-1]).	517,537
**Severe illness.** No potentially life-threatening illness in the period [t_0-365, t_0-1] (Table 4).	408,467
**Substance abuse disorder.** No diagnosed substance abuse disorder (including alcohol) or buprenorphine prescription in [t_0-365, t_0-1].	400,924

[a]Thus, for all patients meeting the first criterion, we reviewed Medicare data from 2012 to identify new users.
[b]If the patient was discharged from the hospital into a skilled nursing facility, the hospital stay included that of the subsequent SNF stay.

D. Severe Illnesses

Because the primary study endpoint is death out of the hospital, we excluded patients for whom such deaths were likely to be related to life-threatening pre-existing illnesses. This is particularly important for hypnotics because failure to adequately control for the life-threatening illnesses that both lead to hypnotic prescriptions and increased risk of death has led most to discount the strong association of hypnotics with increased risk of death.[1,5]

The cohort excluded patients with the life-threatening illnesses listed in Table D. These diseases were identified from medical care encounters in the past year, including hospitalizations, emergency department (ED) and outpatient visits, and in some cases medication prescriptions. A single hospital discharge or ED visit was sufficient for exclusion, as was a single outpatient encounter on t_0-1, the day of the hypnotic prescription fill (hypnotics occasionally started on the day of diagnosis of a severe illness). However, for [t_0-365, t_0-2], at least two outpatient encounters on separate days were required.

Appendix Table D. Severe illness exclusions.

Disease	Remarks
Active cancer	Cancer diagnosis/related procedure or chemotherapy. Cancer diagnoses/procedures must be present for an inpatient or ED encounter in the past year or one of the two required outpatient encounters must occur in [t_0-90,t_0-1]). The diagnoses includes neoplasms of uncertain behavior but do not include skin cancer (except melanoma), carcinoma in situ, neurofibromatosis, polycythemia vera, lymphoproliferative disease, or history of cancer.
HIV/immune deficiencies	Diagnosis or antiretroviral agents appropriate for HIV and pentamidine. Immune deficiencies, graft-vs-host disease
Renal	Chronic renal failure Stage V or end-stage. Includes outpatient dialysis. Also includes patients with ESRD as basis of Medicare enrollment.
Cardio-respiratory	Primary pulmonary hypertension, chronic respiratory failure, cardio-respiratory failure, or pulmonary heart disease (except pulmonary embolus) cardiovascular congenital anomalies. Tracheostomy (excluding temporary) or home ventilator.
Organ transplant	Includes kidney, heart, lung, liver, bone marrow, and pancreas.
Neuromuscular	Cerebral palsy, muscular dystrophy, multiple sclerosis, ALS, Huntington's chorea, quadriplegia, paraplegia, hemiplegia, spinal cord injury
Hematologic	Sickle-cell and other hereditary hemolytic anemias, aplastic anemia.
Gastrointestinal	Liver disease: acute and subacute necrosis of the liver, chronic liver disease and cirrhosis, hepatic encephalopathy, portal hypertension, hepatorenal syndrome, other sequelae of chronic liver disease.
Feeding problems	Outside of hospital: a. Total parenteral nutrition, PEG, enteral feeding, malnutrition; b) Gastrostomy/gastrostomy tube; c) Enterostomy/jejunostomy; d) Esophagus--stricture and stenosis, perforation, esophagostomy; e) Gastrostomy complications; f. Gastrointestinal dilation.
Life-threatening infections	Meningicoccal infections, septicemia, gas gangrene, disseminated candidiasis
Schizophrenia/other psychosis	Either diagnosis or filled prescription for clozapine or long-acting antipsychotic.
Non-specific indicators end-stage disease	Vegetative state, debility, cachexia, gangrene, bedbound, do not resuscitate order.

E. Followup person-time

All study use and followup is restricted to current use of study hypnotics. Fig A provides examples for three hypothetical cohort members and the first 180 days of followup.

Appendix Fig A. Hypnotic use patterns for 3 hypothetical cohort members.

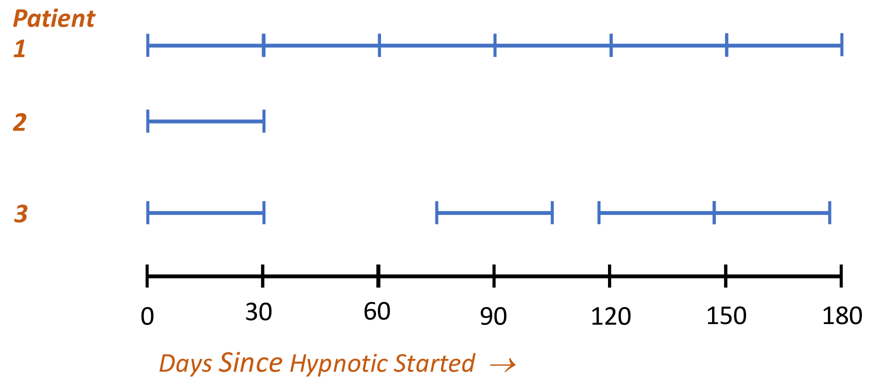

1. Patient fills hypnotic prescriptions without gaps. The analysis includes the entire 180 days of followup.
2. Patient fills a single hypnotic prescription with 30 days of supply. Only these 30 days of person-time are included in the analysis.
3. Patient fills four prescriptions each with 30 days of supply. The followup period includes these 120 days of current hypnotic use.

F. Endpoints

In-hospital deaths were those that occurred during the hospital stay. For patients who were discharged to a skilled nursing facility (SNF), this period included the subsequent SNF stay.

Deaths were classified according to the underlying cause of death, as specified in Table E.

Appendix Table E. Underlying cause of death categories.

ICD 10 Codes	Rubric
Cardiovascular	
E10-E14	Diabetes mellitus[a]
I00-I02	Acute rheumatic fever
I05-I09	Chronic rheumatic heart disease
I10-I15	Hypertensive diseases
I20-I25	Ischemic heart disease
I26-I28	Diseases of pulmonary circulation
I30-I52	Other forms of heart disease
I60-I69	Cerebrovascular disease
I70-I79	Diseases of arteries, arterioles, and capillaries
I80-I89	Diseases of veins, lymphatic vessels and lymph nodes, not elsewhere classified
I95-I99	Other and unspecified disorders of the circulatory system
R570	Cardiogenic shock
R96.0	Instantaneous death
R96.1	Death in <24 hours
R98	Unattended death
R99	Unknown cause
Neurologic	
F00.x-G99.x	Neurologic
Respiratory	
Jxx.x	Diseases of the respiratory system
R05	Cough
R06.x	Abnormalities of breathing
R09.0	Asphyxia
R09.1	Pleurisy
R09.2	Respiratory arrest
W78	Aspiration
Injuries	
Vxx.x, Wxx.x, Xxx.x, Yxx.x	All injuries
X60-X64, Y10-Y14, X40-X44, Y40-Y57	Drug overdose
Cancer	
C00.x-D48.x	Neoplasms

[a]Excludes pregnancy-related diabetes.

G. Covariates

Appendix Table F. Study covariates at baseline according to hypnotic class and baseline opioid use.

a. Benzodiazepines vs trazodone

	BaseOpioid					
	0			1		
	Benzo	Trazodone	sd	Benzo	Trazodone	sd
N	152,711	62,035	.	21,805	9,492	.
Female	70.3%	67.5%	6.1%	68.8%	67.2%	3.5%
White race	87.2%	82.6%	13.0%	85.5%	81.5%	10.6%
Age, years	76.1	76.6	6.8%	75.6	75.6	0.2%
Entered cohort 2014	57.6%	54.1%	7.1%	58.3%	56.5%	3.6%
Medicaid	15.7%	22.4%	17.0%	25.5%	35.3%	21.4%
Mood disorder	13.5%	19.4%	16.0%	16.6%	23.0%	16.0%
Anxiety, panic disorder, or PTSD	8.7%	5.8%	11.2%	8.6%	6.9%	6.2%
Sleep problem, insomnia	19.4%	47.4%	62.1%	21.4%	42.9%	47.2%
Alzheimers and other dementias	8.3%	13.4%	16.7%	6.3%	8.7%	9.0%
Other cognitive impairment	4.5%	7.3%	11.8%	3.2%	4.9%	8.7%
Parkinsons and other movement disorders	5.4%	4.8%	3.1%	5.2%	5.5%	1.4%
Antipsychotic or mood stabilizer	3.1%	4.2%	6.3%	2.9%	3.2%	1.8%
Gabapentinoids/carbamazepine	9.8%	11.6%	5.8%	23.6%	28.8%	11.8%
SSRIs	19.3%	20.0%	1.7%	20.8%	22.8%	4.8%
Other antidepressant	10.4%	14.2%	11.4%	17.2%	22.2%	12.5%
Alzheimer's medications	7.0%	11.1%	14.3%	5.2%	7.0%	7.5%
Parkinson's medications	4.0%	4.1%	0.3%	5.5%	5.7%	1.0%
Cardiac valve disorder	15.4%	13.2%	6.3%	14.6%	12.3%	6.8%
Myocardial infarction	3.9%	4.1%	1.0%	4.9%	5.3%	1.8%
Angina	4.9%	4.0%	4.5%	5.9%	4.8%	4.8%
CABG	4.1%	4.1%	0.0%	4.9%	4.8%	0.4%
Percutaneous intervention	4.2%	4.0%	1.3%	5.2%	5.2%	0.1%
Cardiomyopathy	3.2%	3.0%	0.7%	3.6%	3.2%	2.4%
Conduction disorder	4.8%	4.9%	0.7%	4.9%	4.7%	0.8%
Arrhythmia: atrial fibrillation/flutter	13.2%	12.0%	3.6%	13.5%	12.2%	3.9%
Arrhythmia, except atrial fibrillation	14.3%	12.9%	4.1%	13.6%	12.5%	3.2%
Heart failure	8.8%	9.6%	2.6%	12.2%	13.2%	3.0%
Stroke/intracranial bleed	11.3%	10.6%	2.4%	11.3%	10.9%	1.2%
TIA	3.0%	2.8%	1.1%	2.9%	2.7%	0.9%
Cerebrovascular disease: other or late effects	4.3%	4.9%	3.0%	4.2%	4.7%	2.5%
Peripheral vascular disease	16.3%	16.1%	0.8%	19.5%	19.4%	0.4%
Venous disorders: phlebitis, thrombosis, varico	7.5%	6.7%	2.8%	10.5%	9.2%	4.3%
Diabetes	30.8%	33.0%	4.8%	36.5%	38.1%	3.3%
Obesity	11.2%	12.3%	3.5%	15.6%	17.1%	3.9%
Smoking and smoking-related disorders	9.4%	11.8%	7.8%	14.7%	19.1%	11.7%
Cardiac symptoms	12.2%	9.3%	9.3%	10.5%	8.4%	7.1%
Chronic kidney disease	10.5%	12.6%	6.6%	13.5%	16.0%	7.1%
Angiotensin converting enzyme inhibitors	28.8%	32.7%	8.3%	33.9%	38.6%	9.9%
Angiotensin receptor blocker	26.2%	23.9%	5.2%	24.9%	22.9%	4.7%
Nitrate or other anti-anginal	7.0%	7.1%	0.2%	9.8%	10.1%	0.9%
Anti-arrhythmics	4.0%	2.8%	6.5%	3.8%	2.6%	6.7%
Anticoagulants	9.7%	9.4%	0.9%	11.6%	11.8%	0.6%
Antihypertensives, Other	5.9%	5.6%	1.0%	7.3%	7.4%	0.4%
P2Y12 inhibitor or other antiplatelet drug	9.2%	9.0%	0.7%	11.6%	11.7%	0.3%
Beta-blockers	39.9%	38.9%	2.0%	42.0%	44.0%	4.0%
Calcium channel blockers	29.0%	29.5%	1.0%	31.4%	33.7%	4.9%
Diuretics, Loop	12.8%	14.7%	5.5%	20.9%	23.5%	6.3%
Diuretics, Thiazide	22.9%	22.7%	0.7%	23.7%	23.6%	0.2%
Diuretics, Potassium Sparing (and with hydrochl	6.9%	6.6%	1.0%	8.1%	7.6%	1.9%
Hypoglycemics, Insulin	4.7%	6.5%	7.9%	7.6%	9.5%	6.9%

Hypoglycemics, Metformin	13.5%	16.6%	8.7%	16.7%	18.0%	3.3%
Hypoglycemic, other	10.5%	12.4%	6.0%	13.6%	14.6%	2.9%
Lipid-lowering Drugs, Statins	55.1%	56.3%	2.5%	54.1%	55.4%	2.7%
Lipid-lowering Drugs, Other	9.1%	8.8%	1.3%	10.7%	10.1%	2.0%
Pneumonia	3.5%	3.6%	0.9%	4.1%	4.3%	0.9%
Chronic obstructive pulmonary disease	11.7%	12.8%	3.3%	18.2%	21.4%	8.2%
Asthma	8.5%	8.0%	1.9%	10.2%	10.2%	0.1%
Sleep apnea	7.0%	8.0%	4.0%	8.8%	8.9%	0.6%
Asphyxia or hypoxemia	1.6%	1.9%	2.6%	2.4%	2.9%	2.9%
Home oxygen	4.3%	5.0%	3.4%	7.2%	8.6%	5.4%
Continuous positive airway pressure	4.3%	4.6%	1.5%	5.0%	5.4%	1.6%
Beta-agonists	11.5%	12.7%	3.6%	16.0%	19.0%	7.8%
Bronchodilators, other	5.4%	6.0%	2.5%	7.4%	8.9%	5.7%
Asthma treatment	5.1%	4.6%	2.2%	5.6%	5.7%	0.3%
Inhaled corticosteroids	20.1%	20.6%	1.2%	22.1%	23.2%	2.6%
Unintentional fall (not vigorous activity)	7.0%	9.1%	7.5%	10.5%	12.3%	5.7%
Limited mobility, cane or walker	2.3%	2.7%	2.5%	3.8%	4.8%	5.3%
Wheelchair, hospital bed, or difficulty transfers	2.4%	3.3%	5.7%	4.8%	6.1%	6.0%
Incontinence	6.2%	8.1%	7.5%	7.2%	8.0%	3.4%
Malnutrition/abnormal weight loss/feeding problem/dysphagia	7.4%	8.9%	5.4%	9.1%	10.7%	5.4%
Chronic skin ulcer	2.9%	3.0%	0.8%	4.7%	4.4%	1.2%
Debility, not specified	0.9%	1.3%	4.3%	1.3%	1.8%	3.7%
Neuropathic pain	21.2%	20.7%	1.2%	36.7%	36.8%	0.2%
Fibromyalgia	7.0%	6.9%	0.1%	12.3%	13.5%	3.5%
Back pain/degenerative back disorders	37.4%	36.6%	1.7%	61.8%	64.2%	5.0%
Headache, including migraine	10.2%	10.2%	0.2%	12.6%	12.5%	0.6%
NSAIDs, non-selective	19.8%	21.5%	4.3%	29.8%	31.3%	3.2%
Coxibs	3.6%	3.2%	2.1%	6.0%	5.2%	3.3%
Cyclobenzaprine or other skeletal muscle relaxant	6.3%	6.8%	2.2%	15.9%	18.2%	6.1%
DMARDs	5.2%	5.2%	0.2%	6.9%	6.5%	1.8%
Systemic corticosteroids	19.0%	18.4%	1.5%	26.9%	25.5%	3.2%
Osteoporosis	15.6%	14.8%	2.2%	16.9%	16.0%	2.6%
Osteoporosis medications	8.7%	9.2%	1.6%	9.1%	9.5%	1.2%
Other fracture	5.6%	6.0%	1.7%	9.4%	9.8%	1.5%
Joint replacement	4.9%	5.2%	1.7%	8.5%	9.3%	3.0%
Anemia/transfusion	21.5%	20.5%	2.6%	25.3%	24.9%	0.9%
Prophylactic vaccination	67.5%	67.8%	0.8%	63.7%	64.0%	0.8%
Fluoroquinolones	21.7%	20.4%	3.1%	26.2%	24.6%	3.6%
New psychiatric/neurologic diagnosis	17.4%	43.7%	59.4%	17.4%	38.4%	48.1%
New psychiatric/neurologic prescription	7.4%	9.6%	7.7%	9.5%	11.5%	6.5%
New cardiovascular diagnosis	23.7%	23.1%	1.2%	26.1%	25.6%	1.2%
New cardiovascular prescription	14.6%	15.5%	2.7%	16.1%	17.2%	3.0%
New respiratory diagnosis	6.5%	7.4%	3.7%	8.0%	8.7%	2.8%
New respiratory prescription	6.4%	6.9%	1.9%	6.9%	8.1%	4.8%
New Pain diagnosis	13.6%	13.4%	0.5%	21.1%	20.3%	1.9%
New pain prescription	10.3%	10.1%	0.4%	16.5%	15.1%	4.0%
New frailty diagnosis	7.1%	9.0%	7.3%	10.8%	12.2%	4.4%
Inpatient discharge	1.8%	2.3%	4.0%	3.1%	3.7%	3.3%
Emergency department visit	9.9%	10.9%	3.5%	15.1%	15.4%	0.8%
Home health visit	4.3%	5.6%	6.0%	7.8%	9.1%	4.6%
Outpatient visit day hypnotic prescription fill	40.3%	66.7%	54.9%	47.9%	70.1%	46.4%
Opioid, long-acting	0.0%	0.0%	.	4.8%	5.7%	4.0%
Opioid started past 90 days	0.0%	0.0%	.	25.1%	13.9%	28.4%
Opioid dose 30-59 MME	0.0%	0.0%	.	30.6%	29.6%	2.3%
Opioid dose 60+ MME	0.0%	0.0%	.	29.8%	34.6%	10.4%

b. z-drugs vs trazodone

	BaseOpioid					
	0			1		
	z-drug	Trazodone	sd	z-drug	Trazodone	sd
N	134,359	62,035	.	20,522	9,492	.
Female	60.4%	67.5%	14.9%	60.2%	67.2%	14.6%
White race	84.9%	82.6%	6.4%	84.4%	81.5%	7.5%
Age, years	74.4	76.6	31.2%	74.0	75.6	22.7%
Entered cohort 2014	59.0%	54.1%	9.8%	59.4%	56.5%	5.8%
Medicaid	14.7%	22.4%	19.8%	23.5%	35.3%	26.1%
Mood disorder	10.1%	19.4%	26.5%	13.6%	23.0%	24.3%
Anxiety, panic disorder, or PTSD	4.3%	5.8%	7.0%	5.2%	6.9%	7.1%
Sleep problem, insomnia	36.0%	47.4%	23.3%	32.7%	42.9%	21.1%
Alzheimers and other dementias	3.8%	13.4%	34.8%	3.9%	8.7%	19.6%
Other cognitive impairment	3.0%	7.3%	19.8%	2.6%	4.9%	12.2%
Parkinsons and other movement disorders	3.2%	4.8%	7.8%	3.9%	5.5%	7.4%
Antipsychotic or mood stabilizer	1.6%	4.2%	15.7%	1.9%	3.2%	8.6%
Gabapentinoids/carbamazepine	9.5%	11.6%	6.9%	22.8%	28.8%	13.6%
SSRIs	14.1%	20.0%	15.8%	17.5%	22.8%	13.1%
Other antidepressant	9.6%	14.2%	14.3%	16.1%	22.2%	15.4%
Alzheimer's medications	3.5%	11.1%	29.5%	3.6%	7.0%	15.6%
Parkinson's medications	2.7%	4.1%	7.7%	4.0%	5.7%	7.8%
Cardiac valve disorder	14.0%	13.2%	2.4%	13.1%	12.3%	2.7%
Myocardial infarction	3.5%	4.1%	3.3%	5.1%	5.3%	0.9%
Angina	4.6%	4.0%	2.8%	5.2%	4.8%	1.5%
CABG	4.0%	4.1%	0.9%	4.8%	4.8%	0.1%
Percutaneous intervention	4.1%	4.0%	0.7%	5.3%	5.2%	0.4%
Cardiomyopathy	3.1%	3.0%	0.4%	3.5%	3.2%	1.9%
Conduction disorder	4.7%	4.9%	1.0%	4.8%	4.7%	0.6%
Arrhythmia: atrial fibrillation/flutter	11.8%	12.0%	0.5%	12.1%	12.2%	0.4%
Arrhythmia, except atrial fibrillation	13.0%	12.9%	0.5%	13.4%	12.5%	2.7%
Heart failure	7.6%	9.6%	7.1%	10.4%	13.2%	8.5%
Stroke/intracranial bleed	9.7%	10.6%	2.8%	10.2%	10.9%	2.3%
TIA	2.4%	2.8%	2.6%	2.2%	2.7%	3.1%
Cerebrovascular disease: other or late effects	3.2%	4.9%	8.5%	3.7%	4.7%	5.1%
Peripheral vascular disease	14.1%	16.1%	5.4%	17.9%	19.4%	3.9%
Venous disorders: phlebitis, thrombosis, varico	6.4%	6.7%	1.4%	8.7%	9.2%	2.0%
Diabetes	29.4%	33.0%	7.9%	35.8%	38.1%	4.8%
Obesity	11.5%	12.3%	2.7%	16.4%	17.1%	1.9%
Smoking and smoking-related disorders	9.4%	11.8%	7.6%	16.0%	19.1%	8.0%
Cardiac symptoms	10.8%	9.3%	5.0%	10.2%	8.4%	6.1%
Chronic kidney disease	9.0%	12.6%	11.5%	12.1%	16.0%	11.2%
Angiotensin converting enzyme inhibitors	27.4%	32.7%	11.5%	33.0%	38.6%	11.7%
Angiotensin receptor blocker	25.3%	23.9%	3.4%	24.1%	22.9%	2.9%
Nitrate or other anti-anginal	6.2%	7.1%	3.3%	8.6%	10.1%	5.2%
Anti-arrhythmics	3.8%	2.8%	5.4%	3.9%	2.6%	7.0%
Anticoagulants	9.5%	9.4%	0.2%	11.8%	11.8%	0.0%
Antihypertensives, Other	4.8%	5.6%	3.8%	6.6%	7.4%	3.0%
P2Y12 inhibitor or other antiplatelet drug	8.3%	9.0%	2.4%	10.6%	11.7%	3.5%
Beta-blockers	34.6%	38.9%	9.1%	38.2%	44.0%	11.8%
Calcium channel blockers	26.0%	29.5%	7.7%	28.5%	33.7%	11.2%
Diuretics, Loop	10.5%	14.7%	12.5%	17.3%	23.5%	15.3%
Diuretics, Thiazide	21.5%	22.7%	2.7%	23.0%	23.6%	1.4%
Diuretics, Potassium Sparing (and with hydrochl	6.1%	6.6%	2.2%	7.2%	7.6%	1.7%
Hypoglycemics, Insulin	4.5%	6.5%	8.8%	7.2%	9.5%	8.3%
Hypoglycemics, Metformin	14.0%	16.6%	7.1%	17.1%	18.0%	2.3%

Hypoglycemic, other	10.4%	12.4%	6.3%	13.5%	14.6%	3.1%
Lipid-lowering Drugs, Statins	56.9%	56.3%	1.2%	55.8%	55.4%	0.6%
Lipid-lowering Drugs, Other	9.7%	8.8%	3.2%	10.8%	10.1%	2.2%
Pneumonia	3.2%	3.6%	2.4%	3.8%	4.3%	2.6%
Chronic obstructive pulmonary disease	10.0%	12.8%	9.0%	16.2%	21.4%	13.5%
Asthma	8.6%	8.0%	2.3%	10.5%	10.2%	1.0%
Sleep apnea	10.0%	8.0%	6.7%	10.3%	8.9%	4.6%
Asphyxia or hypoxemia	1.6%	1.9%	2.6%	2.1%	2.9%	5.2%
Home oxygen	4.0%	5.0%	4.6%	6.1%	8.6%	9.6%
Continuous positive airway pressure	5.6%	4.6%	4.5%	5.9%	5.4%	2.3%
Beta-agonists	11.2%	12.7%	4.6%	15.7%	19.0%	8.8%
Bronchodilators, other	5.4%	6.0%	2.5%	7.3%	8.9%	5.9%
Asthma treatment	5.3%	4.6%	3.2%	5.8%	5.7%	0.5%
Inhaled corticosteroids	21.4%	20.6%	2.1%	23.0%	23.2%	0.4%
Unintentional fall (not vigorous activity)	5.4%	9.1%	14.1%	9.2%	12.3%	10.2%
Limited mobility, cane or walker	2.3%	2.7%	3.1%	4.4%	4.8%	1.9%
Wheelchair, hospital bed, or difficulty transfers	1.8%	3.3%	9.5%	4.0%	6.1%	9.7%
Incontinence	5.7%	8.1%	9.6%	6.6%	8.0%	5.6%
Malnutrition/abnormal weight loss/feeding problem/dysphagia	6.1%	8.9%	10.6%	7.6%	10.7%	10.7%
Chronic skin ulcer	2.2%	3.0%	5.0%	3.8%	4.4%	2.8%
Debility, not specified	0.6%	1.3%	7.8%	1.1%	1.8%	5.9%
Neuropathic pain	21.5%	20.7%	1.8%	36.5%	36.8%	0.6%
Fibromyalgia	7.0%	6.9%	0.4%	12.5%	13.5%	2.8%
Back pain/degenerative back disorders	37.5%	36.6%	1.9%	61.6%	64.2%	5.4%
Headache, including migraine	9.0%	10.2%	4.4%	11.6%	12.5%	2.7%
NSAIDs, non-selective	21.5%	21.5%	0.0%	32.3%	31.3%	2.2%
Coxibs	5.0%	3.2%	8.9%	7.9%	5.2%	10.8%
Cyclobenzaprine or other skeletal muscle relaxant	6.5%	6.8%	1.3%	16.3%	18.2%	5.0%
DMARDs	5.9%	5.2%	3.4%	7.0%	6.5%	1.9%
Systemic corticosteroids	19.1%	18.4%	1.8%	26.7%	25.5%	2.8%
Osteoporosis	14.4%	14.8%	1.1%	14.5%	16.0%	4.2%
Osteoporosis medications	8.9%	9.2%	0.8%	8.8%	9.5%	2.3%
Other fracture	5.3%	6.0%	3.0%	9.0%	9.8%	2.7%
Joint replacement	5.8%	5.2%	2.5%	10.7%	9.3%	4.6%
Anemia/transfusion	19.5%	20.5%	2.4%	23.7%	24.9%	2.8%
Prophylactic vaccination	70.4%	67.8%	5.6%	64.5%	64.0%	0.9%
Fluoroquinolones	21.5%	20.4%	2.6%	24.7%	24.6%	0.3%
New psychiatric/neurologic diagnosis	26.5%	43.7%	36.6%	24.2%	38.4%	30.9%
New psychiatric/neurologic prescription	5.2%	9.6%	16.6%	8.3%	11.5%	10.8%
New cardiovascular diagnosis	21.8%	23.1%	3.1%	25.7%	25.6%	0.3%
New cardiovascular prescription	13.9%	15.5%	4.6%	16.0%	17.2%	3.5%
New respiratory diagnosis	8.1%	7.4%	2.3%	8.5%	8.7%	0.9%
New respiratory prescription	6.9%	6.9%	0.0%	6.8%	8.1%	5.0%
New Pain diagnosis	13.0%	13.4%	1.4%	20.2%	20.3%	0.2%
New pain prescription	11.1%	10.1%	3.2%	17.4%	15.1%	6.2%
New frailty diagnosis	5.9%	9.0%	11.8%	10.4%	12.2%	5.6%
Inpatient discharge	2.2%	2.3%	1.0%	4.6%	3.7%	4.3%
Emergency department visit	7.4%	10.9%	12.2%	13.0%	15.4%	6.7%
Home health visit	3.0%	5.6%	13.0%	6.7%	9.1%	8.8%
Outpatient visit day hypnotic prescription fill	41.0%	66.7%	53.4%	50.1%	70.1%	41.7%
Opioid, long-acting	0.0%	0.0%	.	4.3%	5.7%	6.7%
Opioid started past 90 days	0.0%	0.0%	.	27.1%	13.9%	33.0%
Opioid dose 30-59 MME	0.0%	0.0%	.	33.3%	29.6%	8.0%
Opioid dose 60+ MME	0.0%	0.0%	.	30.4%	34.6%	9.0%

H. Propensity Score

Covariates. Because patient characteristics and comorbidity were likely to vary according to both specific hypnotic and opioid use, the analysis controlled for numerous covariates. There were two types of covariates, those that described *patient characteristics and comorbidity* and those related to *current opioid use*. The covariates. The patient characteristics included 101 covariates plausibly associated with both risk of death and the use of specific hypnotics or opioids (Appendix Table F). They were defined from medical care encounters in the preceding year and included psychiatric and neurologic disorders, cardiovascular and renal conditions, respiratory diseases, pain-related diagnoses and medications, measures of frailty, and medical care utilization. The selection and definition of covariates was primarily based on our previous studies of out-of-hospital death,[6,7] which included variables based on standard measures of comorbidity.[8,9] We also reviewed the literature to add several indicators of frailty.[10] Because changes in comorbidity during followup could be associated with changes in the likelihood of concurrent opioids, the values were updated on the date of each hypnotic prescription fill.

The risk of opioid use may vary according to duration of action, time since initiation of treatment, and opioid dose. Thus, we controlled for four opioid-related covariates.

Long-acting opioid, see Appendix Table B.
New use of opioids, defined as initiation within the past 90 days.
Intermediate dose, defined as 30-59 morphine-milligram equivalents (Appendix Table B).
High dose, defined as ≥60 morphine-milligram equivalents (Appendix Table B).

Because these covariates could change on any given day (initiate opioid use during the period of days of supply for a hypnotic), these were updated on a daily basis.

Propensity score definition and rationale. The propensity score is the probability that a patient receives one of two treatment options, conditional on the study covariates. If the propensity score is properly calculated, controlling for the propensity score in the analysis is equivalent to adjusting for all of the variables that go into its calculation.[11-13] The key advantage of propensity scores is the ability to control for much larger numbers of covariates than traditional multivariate methods, which require 7-10 endpoints for every variable in the model. As Haukoos and Lewis note in a review:[13]

> *"Propensity score methods generally allow many more variables to be included in the propensity score model, which increases the ability of these approaches to effectively adjust for confounding, than could be incorporated directly into a multivariable analysis of the study outcome."*

Assessing balance. A properly formulated propensity score is a balancing score, that is, the distribution of the covariates conditional on the propensity score is the same in both the treated and untreated groups. Balance is measured by examining the distribution of the covariates in each treatment group after inverse-odds-of-treatment (IOT) weighting[a] and calculating the standardized difference,[12] with a difference of less than 10% considered good balance.[13]

Propensity score calculation. Because both the use of opioids and patient characteristics could change during followup, the study propensity score was time-dependent, with covariate values updated at the time of each study hypnotic prescription fill.[14] Three propensity scores were calculated for each of the pairwise comparisons of study hypnotics: trazodone vs benzodiazepines, trazodone vs z-drugs, and benzodiazepines vs z-drugs. The propensity score was estimated with logistic regression models with SAS version 9 PROC LOGISTIC. These included all of the covariates in Appendix Table F except for the opioid use characteristics covariates, which were included directly in the proportional hazards regression. Because the factors that predicted individual hypnotic use might differ depending on opioid use, separate regression models were fit for users and nonusers of opioids, which assured covariate balance for each of these groups.

---

[a] More commonly, inverse probability of treatment weighting is used. However, because our study estimates the average treatment effect in the treated (equivalent to matching trazodone users to those of other hypnotics), the appropriate weighting is IOT.

Use in analysis. There are four standard methods for analysis with propensity scores. Cohort members can be *matched* according to the propensity score, each observation can be assigned a *weight* according to the propensity score value, the analysis can be *stratified* by quantiles of the propensity score, or the propensity score can be included in a *regression model* for the study endpoint.[11-13] The first three methods are the most commonly used because regression modelling requires specifying the functional form of the relation between the propensity score and the endpoint, which depends on additional assumptions.

We considered both matching and weighting, as these methods are the least subject to residual confounding.[12] However, matching is both more complex with time-dependent exposures and reduces study power by discarding observations. Weighted analyses typically use weights inversely proportional to the propensity score, which commonly leads to large weights that inflate variances and consequently reduce power. Thus, the primary analysis used stratification by the deciles of the time-dependent propensity score. To assess the potential for residual confounding, sensitivity analyses were performed with both matching and weighting.

The strata were defined according to the distribution of the propensity score in the trazodone group. This procedure estimates the average treatment effect in the treated, the same estimate that would result from matching each trazodone user with either a benzodiazepine or z-drug user.[12]

We did not include the opioid-related covariates in the propensity score because these could change on any day of followup whereas the patient characteristics were updated only at the time of each prescription fill. Our rationale was that the patient comorbidity changes during the span of a prescription (for example, a fall or a diagnosis of dementia) could be precursors of an endpoint. Furthermore, the theory on which use of a time-dependent propensity score is based[14] assumes covariates are updated no more frequently than at each prescription fill. Thus, the opioid-related covariates were controlled for directly in the regression model.

Balance assessment. Appendix Table F shows the distribution of the covariates and the standardized differences (relative to trazodone), with standardized differences greater than 10% for many of the covariates. Appendix Table G shows the distribution and standardized differences after IOT weighting; all differences were <2%, indicating good balance.

Appendix Table G. Study covariates at baseline according to hypnotic class and baseline opioid use, weighted according to inverse odds of treatment. Opioid-related covariates, which varied on each person-day of followup, were not included in the propensity score because they were directly controlled for in the analysis.

a. Benzodiazepines vs trazodone

	BaseOpioid					
	0			1		
	BaseHypnotic			BaseHypnotic		
	Benzo	Trazodone	sd	Benzo	Trazodone	sd
N of prescriptions	152,711	62,035		21,805	9,492	
Age, years	76.7	76.6	1.3%	75.7	75.6	1.3%
	7.8	7.8		7.5	7.7	
Female	66.6%	67.5%	1.9%	66.6%	67.2%	1.3%
White race	82.1%	82.6%	1.3%	81.3%	81.6%	0.8%
Calendar year	54.1%	54.1%	0.0%	56.9%	56.5%	0.8%
Medicaid	23.1%	22.4%	1.7%	36.0%	35.3%	1.5%
Mood disorder	19.8%	19.4%	1.0%	23.0%	23.0%	0.0%
Anxiety, panic disorder, or PTSD	5.6%	5.8%	0.9%	6.9%	6.9%	0.0%
Sleep problem, insomnia	47.2%	47.4%	0.4%	43.0%	42.9%	0.2%
Alzheimers and other dementias	13.3%	13.4%	0.3%	8.6%	8.7%	0.4%
Other cognitive impairment	7.4%	7.3%	0.4%	4.8%	4.8%	0.0%
Parkinsons and other movement disorders	4.8%	4.8%	0.0%	5.7%	5.5%	0.9%
Antipsychotic or mood stabilizer	4.5%	4.2%	1.5%	3.4%	3.2%	1.1%
Gabapentinoids/carbamazepine	12.0%	11.6%	1.2%	29.4%	28.8%	1.3%
SSRIs	20.6%	20.0%	1.5%	23.1%	22.8%	0.7%
Other antidepressant	14.7%	14.2%	1.4%	23.1%	22.2%	2.2%
Alzheimer's medications	11.4%	11.1%	0.9%	7.1%	7.0%	0.4%
Parkinson's medications	4.3%	4.1%	1.0%	6.0%	5.7%	1.3%
Cardiovascular valve disease	13.3%	13.2%	0.3%	12.0%	12.2%	0.6%
Myocardial infarction	4.2%	4.1%	0.5%	5.6%	5.3%	1.3%
Angina	4.1%	4.0%	0.5%	5.0%	4.8%	0.9%
CABG	4.2%	4.1%	0.5%	5.0%	4.8%	0.9%
Percutaneous intervention	4.1%	4.0%	0.5%	5.3%	5.2%	0.4%
Cardiomyopathy	3.1%	3.0%	0.6%	3.2%	3.1%	0.6%
Conduction disorder	5.0%	4.9%	0.5%	4.6%	4.7%	0.5%
Arrhythmia: atrial fibrillation/flutter	12.2%	12.0%	0.6%	12.4%	12.2%	0.6%
Arrhythmia, except atrial fibrillation	12.9%	12.8%	0.3%	12.5%	12.5%	0.0%
Heart failure	9.9%	9.6%	1.0%	13.4%	13.2%	0.6%
Stroke/intracranial bleed	10.7%	10.6%	0.3%	11.2%	10.9%	1.0%
TIA	2.8%	2.8%	0.0%	2.8%	2.7%	0.6%
Cerebrovascular disease: other or late effects	5.0%	4.9%	0.5%	4.9%	4.7%	0.9%
Peripheral vascular disease	16.4%	16.1%	0.8%	19.5%	19.4%	0.3%
Venous disorders: phlebitis, thrombosis, varicose veins	6.8%	6.7%	0.4%	9.2%	9.2%	0.0%
Diabetes	33.7%	33.0%	1.5%	38.2%	38.1%	0.2%
Obesity	12.5%	12.3%	0.6%	17.1%	17.1%	0.0%
Smoking and smoking-related disorders	11.9%	11.8%	0.3%	19.4%	19.1%	0.8%
Cardiac symptoms	9.3%	9.3%	0.0%	8.4%	8.4%	0.0%
Chronic kidney disease	12.9%	12.6%	0.9%	16.3%	16.0%	0.8%
Angiotensin converting enzyme inhibitors	32.8%	32.7%	0.2%	38.7%	38.6%	0.2%
Angiotensin receptor blocker	23.9%	23.9%	0.0%	22.8%	22.9%	0.2%
Nitrate or other anti-anginal	7.3%	7.1%	0.8%	10.3%	10.1%	0.7%
Anti-arrhythmics	2.9%	2.8%	0.6%	2.8%	2.6%	1.2%
Anticoagulants	9.6%	9.4%	0.7%	11.9%	11.8%	0.3%
Antihypertensives, Other	5.8%	5.6%	0.9%	7.4%	7.4%	0.0%
P2Y12 inhibitor or other antiplatelet drug	9.3%	9.0%	1.0%	12.0%	11.7%	0.9%
Beta-blockers	39.0%	38.9%	0.2%	44.1%	44.0%	0.2%
Calcium channel blockers	29.6%	29.5%	0.2%	33.8%	33.6%	0.4%
Diuretics, Loop	15.2%	14.7%	1.4%	24.0%	23.5%	1.2%

Diuretics, Thiazide	22.5%	22.7%	0.5%	23.6%	23.6%	0.0%
Diuretics, Potassium Sparing (and with hydrochlorothiazide)	6.6%	6.6%	0.0%	7.7%	7.6%	0.4%
Hypoglycemics, Insulin	6.8%	6.5%	1.2%	9.6%	9.5%	0.3%
Hypoglycemics, Metformin	16.9%	16.6%	0.8%	17.9%	18.0%	0.3%
Hypoglycemic, other	12.8%	12.4%	1.2%	14.7%	14.6%	0.3%
Lipid-lowering Drugs, Statins	56.6%	56.3%	0.6%	55.7%	55.4%	0.6%
Lipid-lowering Drugs, Other	9.0%	8.8%	0.7%	10.2%	10.1%	0.3%
Pneumonia	3.7%	3.6%	0.5%	4.4%	4.3%	0.5%
Chronic obstructive pulmonary disease	13.0%	12.8%	0.6%	22.1%	21.4%	1.7%
Asthma	8.0%	8.0%	0.0%	10.0%	10.2%	0.7%
Sleep apnea	8.4%	8.0%	1.5%	9.0%	8.9%	0.4%
Asphyxia or hypoxemia	2.1%	1.9%	1.4%	2.9%	2.9%	0.0%
Home oxygen	5.2%	5.0%	0.9%	8.8%	8.6%	0.7%
Continuous positive airway pressure	4.8%	4.6%	0.9%	5.4%	5.3%	0.4%
Beta-agonists	12.7%	12.7%	0.0%	19.1%	19.0%	0.3%
Bronchodilators, other	5.9%	6.0%	0.4%	9.0%	8.9%	0.4%
Asthma treatment	4.7%	4.6%	0.5%	6.0%	5.7%	1.3%
Inhaled corticosteroids	20.6%	20.6%	0.0%	23.2%	23.1%	0.2%
Unintentional fall (not vigorous activity)	9.2%	9.1%	0.3%	12.4%	12.3%	0.3%
Limited mobility, cane or walker	2.8%	2.7%	0.6%	4.9%	4.8%	0.5%
Wheelchair, hospital bed, or difficulty transfers	3.5%	3.3%	1.1%	6.2%	6.1%	0.4%
Incontinence	8.2%	8.1%	0.4%	8.1%	8.1%	0.0%
Malnutrition/abnormal weight loss/feeding problem/dysphagia	9.0%	8.9%	0.4%	11.1%	10.7%	1.3%
Chronic skin ulcer	3.1%	3.0%	0.6%	4.5%	4.4%	0.5%
Debility, not specified	1.4%	1.3%	0.9%	1.8%	1.8%	0.0%
Neuropathic pain	21.0%	20.7%	0.7%	37.3%	36.8%	1.0%
Fibromyalgia	7.1%	6.9%	0.8%	13.6%	13.5%	0.3%
Back pain/degenerative back disorders	36.7%	36.6%	0.2%	64.4%	64.2%	0.4%
Headache, including migraine	10.3%	10.2%	0.3%	12.7%	12.5%	0.6%
NSAIDs, non-selective	22.0%	21.5%	1.2%	31.5%	31.2%	0.6%
Coxibs	3.4%	3.2%	1.1%	5.3%	5.2%	0.4%
Cyclobenzaprine or other skeletal muscle relaxant	6.9%	6.8%	0.4%	18.3%	18.2%	0.3%
DMARDs	5.3%	5.2%	0.4%	6.6%	6.5%	0.4%
PainRx_Steroids	18.7%	18.4%	0.8%	25.5%	25.5%	0.0%
Osteoporosis	14.9%	14.8%	0.3%	16.1%	16.0%	0.3%
Osteoporosis medications	9.3%	9.2%	0.3%	9.7%	9.5%	0.7%
Other fracture	6.1%	6.0%	0.4%	10.3%	9.8%	1.7%
Joint replacement	5.2%	5.2%	0.0%	9.2%	9.3%	0.3%
Anemia/transfusion	20.8%	20.4%	1.0%	25.3%	24.8%	1.2%
Prophylactic vaccination	67.7%	67.8%	0.2%	63.8%	64.0%	0.4%
Fluoroquinolones	20.7%	20.4%	0.7%	24.8%	24.6%	0.5%
New psychiatric/neurologic diagnosis	43.5%	43.7%	0.4%	38.6%	38.4%	0.4%
New psychiatric/neurologic prescription	10.2%	9.5%	2.3%	12.2%	11.5%	2.2%
New cardiovascular diagnosis	23.4%	23.1%	0.7%	26.0%	25.6%	0.9%
New cardiovascular prescription	15.8%	15.5%	0.8%	17.6%	17.2%	1.1%
New respiratory diagnosis	7.7%	7.4%	1.1%	9.1%	8.7%	1.4%
New respiratory prescription	6.9%	6.9%	0.0%	8.4%	8.1%	1.1%
New Pain diagnosis	13.5%	13.4%	0.3%	20.2%	20.3%	0.2%
New pain prescription	10.2%	10.1%	0.3%	14.9%	15.1%	0.6%
New frailty diagnosis	9.4%	9.0%	1.4%	12.4%	12.2%	0.6%
Inpatient discharge	2.5%	2.3%	1.3%	3.9%	3.7%	1.0%
Emergency department visit	11.2%	10.9%	1.0%	15.4%	15.4%	0.0%
Home health visit	6.0%	5.6%	1.7%	9.5%	9.1%	1.4%
Outpatient visit day hypnotic prescription fill	66.4%	66.7%	0.6%	70.0%	70.1%	0.2%

b. z-Drugs vs trazodone

	BaseOpioid 0			BaseOpioid 1		
	BaseHypnotic			BaseHypnotic		
	z-Drugs	Trazodone	sd	z-Drugs	Trazodone	sd
N of prescriptions	134,359	62,035		20,522	9,492	
Age, years	76.7	76.6	1.3%	75.7	75.6	1.3%
	7.8	7.8		7.6	7.7	
Female	67.4%	67.5%	0.2%	67.3%	67.2%	0.2%
White race	82.2%	82.6%	1.1%	80.6%	81.6%	2.6%
Calendar year	54.2%	54.1%	0.2%	56.5%	56.5%	0.0%
Medicaid	23.1%	22.4%	1.7%	36.5%	35.3%	2.5%
Mood disorder	19.9%	19.4%	1.3%	23.2%	23.0%	0.5%
Anxiety, panic disorder, or PTSD	6.0%	5.8%	0.8%	7.2%	6.9%	1.2%
Sleep problem, insomnia	47.9%	47.4%	1.0%	43.7%	42.9%	1.6%
Alzheimers and other dementias	13.5%	13.4%	0.3%	8.6%	8.7%	0.4%
Other cognitive impairment	7.6%	7.3%	1.1%	4.7%	4.8%	0.5%
Parkinsons and other movement disorders	5.0%	4.8%	0.9%	5.6%	5.5%	0.4%
Antipsychotic or mood stabilizer	4.6%	4.2%	2.0%	3.3%	3.2%	0.6%
Gabapentinoids/carbamazepine	12.0%	11.6%	1.2%	29.5%	28.8%	1.5%
SSRIs	20.7%	20.0%	1.7%	23.2%	22.8%	1.0%
Other antidepressant	15.0%	14.2%	2.3%	22.9%	22.2%	1.7%
Alzheimer's medications	11.6%	11.1%	1.6%	7.1%	7.0%	0.4%
Parkinson's medications	4.4%	4.1%	1.5%	5.9%	5.7%	0.9%
Cardiovascular valve disease	13.2%	13.2%	0.0%	12.2%	12.2%	0.0%
Myocardial infarction	4.2%	4.1%	0.5%	5.4%	5.3%	0.4%
Angina	4.1%	4.0%	0.5%	5.0%	4.8%	0.9%
CABG	4.3%	4.1%	1.0%	4.9%	4.8%	0.5%
Percutaneous intervention	4.2%	4.0%	1.0%	5.3%	5.2%	0.4%
Cardiomyopathy	3.1%	3.0%	0.6%	3.3%	3.1%	1.1%
Conduction disorder	5.0%	4.9%	0.5%	4.6%	4.7%	0.5%
Arrhythmia: atrial fibrillation/flutter	12.1%	12.0%	0.3%	12.4%	12.2%	0.6%
Arrhythmia, except atrial fibrillation	12.9%	12.8%	0.3%	12.6%	12.5%	0.3%
Heart failure	9.9%	9.6%	1.0%	13.7%	13.2%	1.5%
Stroke/intracranial bleed	10.7%	10.6%	0.3%	10.9%	10.9%	0.0%
TIA	2.8%	2.8%	0.0%	2.7%	2.7%	0.0%
Cerebrovascular disease: other or late effects	5.1%	4.9%	0.9%	4.8%	4.7%	0.5%
Peripheral vascular disease	16.4%	16.1%	0.8%	19.5%	19.4%	0.3%
Venous disorders: phlebitis, thrombosis, varicose veins	6.8%	6.7%	0.4%	9.4%	9.2%	0.7%
Diabetes	33.6%	33.0%	1.3%	38.5%	38.1%	0.8%
Obesity	12.5%	12.3%	0.6%	17.1%	17.1%	0.0%
Smoking and smoking-related disorders	12.0%	11.8%	0.6%	19.2%	19.1%	0.3%
Cardiac symptoms	9.4%	9.3%	0.3%	8.5%	8.4%	0.4%
Chronic kidney disease	13.0%	12.6%	1.2%	16.2%	16.0%	0.5%
Angiotensin converting enzyme inhibitors	33.1%	32.7%	0.9%	39.0%	38.6%	0.8%
Angiotensin receptor blocker	24.1%	23.9%	0.5%	22.9%	22.9%	0.0%
Nitrate or other anti-anginal	7.3%	7.1%	0.8%	10.4%	10.1%	1.0%
Anti-arrhythmics	2.9%	2.8%	0.6%	2.7%	2.6%	0.6%
Anticoagulants	9.5%	9.4%	0.3%	12.1%	11.8%	0.9%
Antihypertensives, Other	5.8%	5.6%	0.9%	7.5%	7.4%	0.4%
P2Y12 inhibitor or other antiplatelet drug	9.3%	9.0%	1.0%	12.0%	11.7%	0.9%
Beta-blockers	39.5%	38.9%	1.2%	44.6%	44.0%	1.2%
Calcium channel blockers	29.9%	29.5%	0.9%	34.0%	33.6%	0.8%
Diuretics, Loop	15.4%	14.7%	2.0%	24.5%	23.5%	2.3%
Diuretics, Thiazide	22.7%	22.7%	0.0%	23.8%	23.6%	0.5%
Diuretics, Potassium Sparing (and with hydrochlorothiazide)	6.7%	6.6%	0.4%	7.6%	7.6%	0.0%
Hypoglycemics, Insulin	6.7%	6.5%	0.8%	9.8%	9.5%	1.0%
Hypoglycemics, Metformin	16.9%	16.6%	0.8%	18.4%	18.0%	1.0%
Hypoglycemic, other	12.8%	12.4%	1.2%	15.0%	14.6%	1.1%
Lipid-lowering Drugs, Statins	56.5%	56.3%	0.4%	55.6%	55.4%	0.4%

Lipid-lowering Drugs, Other	8.8%	8.8%	0.0%	10.3%	10.1%	0.7%
Pneumonia	3.8%	3.6%	1.1%	4.4%	4.3%	0.5%
Chronic obstructive pulmonary disease	13.2%	12.8%	1.2%	21.8%	21.4%	1.0%
Asthma	8.1%	8.0%	0.4%	10.2%	10.2%	0.0%
Sleep apnea	8.3%	8.0%	1.1%	9.3%	8.9%	1.4%
Asphyxia or hypoxemia	2.0%	1.9%	0.7%	2.8%	2.9%	0.6%
Home oxygen	5.2%	5.0%	0.9%	9.0%	8.6%	1.4%
Continuous positive airway pressure	4.7%	4.6%	0.5%	5.5%	5.3%	0.9%
Beta-agonists	12.9%	12.7%	0.6%	19.2%	19.0%	0.5%
Bronchodilators, other	6.1%	6.0%	0.4%	9.0%	8.9%	0.4%
Asthma treatment	4.6%	4.6%	0.0%	5.8%	5.7%	0.4%
Inhaled corticosteroids	20.8%	20.6%	0.5%	23.0%	23.1%	0.2%
Unintentional fall (not vigorous activity)	9.4%	9.1%	1.0%	12.3%	12.3%	0.0%
Limited mobility, cane or walker	2.8%	2.7%	0.6%	4.9%	4.8%	0.5%
Wheelchair, hospital bed, or difficulty transfers	3.6%	3.3%	1.6%	6.3%	6.1%	0.8%
Incontinence	8.3%	8.1%	0.7%	8.1%	8.1%	0.0%
Malnutrition/abnormal weight loss/feeding problem/dysphagia	8.9%	8.9%	0.0%	11.2%	10.7%	1.6%
Chronic skin ulcer	3.1%	3.0%	0.6%	4.6%	4.4%	1.0%
Debility, not specified	1.4%	1.3%	0.9%	1.7%	1.8%	0.8%
Neuropathic pain	21.2%	20.7%	1.2%	37.2%	36.8%	0.8%
Fibromyalgia	7.1%	6.9%	0.8%	13.8%	13.5%	0.9%
Back pain/degenerative back disorders	37.0%	36.6%	0.8%	64.6%	64.2%	0.8%
Headache, including migraine	10.4%	10.2%	0.7%	12.6%	12.5%	0.3%
NSAIDs, non-selective	21.7%	21.5%	0.5%	31.6%	31.2%	0.9%
Coxibs	3.3%	3.2%	0.6%	5.2%	5.2%	0.0%
Cyclobenzaprine or other skeletal muscle relaxant	7.0%	6.8%	0.8%	18.4%	18.2%	0.5%
DMARDs	5.2%	5.2%	0.0%	6.7%	6.5%	0.8%
PainRx_Steroids	18.7%	18.4%	0.8%	25.6%	25.5%	0.2%
Osteoporosis	14.8%	14.8%	0.0%	16.0%	16.0%	0.0%
Osteoporosis medications	9.3%	9.2%	0.3%	9.6%	9.5%	0.3%
Other fracture	6.1%	6.0%	0.4%	9.8%	9.8%	0.0%
Joint replacement	5.3%	5.2%	0.4%	9.3%	9.3%	0.0%
Anemia/transfusion	20.8%	20.4%	1.0%	25.3%	24.8%	1.2%
Prophylactic vaccination	67.5%	67.8%	0.6%	63.5%	64.0%	1.0%
Fluoroquinolones	20.6%	20.4%	0.5%	24.6%	24.6%	0.0%
New psychiatric/neurologic diagnosis	44.1%	43.7%	0.8%	39.0%	38.4%	1.2%
New psychiatric/neurologic prescription	10.4%	9.5%	3.0%	12.1%	11.5%	1.9%
New cardiovascular diagnosis	23.3%	23.1%	0.5%	25.5%	25.6%	0.2%
New cardiovascular prescription	16.0%	15.5%	1.4%	17.8%	17.2%	1.6%
New respiratory diagnosis	7.6%	7.4%	0.8%	9.0%	8.7%	1.1%
New respiratory prescription	7.0%	6.9%	0.4%	8.3%	8.1%	0.7%
New Pain diagnosis	13.5%	13.4%	0.3%	20.3%	20.3%	0.0%
New pain prescription	10.2%	10.1%	0.3%	15.1%	15.1%	0.0%
New frailty diagnosis	9.3%	9.0%	1.0%	12.2%	12.2%	0.0%
Inpatient discharge	2.4%	2.3%	0.7%	3.9%	3.7%	1.0%
Emergency department visit	11.4%	10.9%	1.6%	15.7%	15.4%	0.8%
Home health visit	6.0%	5.6%	1.7%	9.4%	9.1%	1.0%
Outpatient visit day hypnotic prescription fill	67.1%	66.7%	0.9%	70.3%	70.1%	0.4%

I. Statistical Analysis

Primary Analysis

The primary analysis was stratified according to deciles of a time-dependent propensity score, with the covariates (excluding those characterizing prescribed opioids) updated at the time of each hypnotic prescription fill. The opioid characteristics were updated for each person-day of followup.

The following SAS program template shows the regression model:

```
PROC PHREG;
    STRATA PS_STRATA; /* note there is a separate variable for each pairwise hypnotic comparison*/

    CLASS HYPOPIOID;
    /* Design variable that specifies the hypnotics and opioid use, reference trazodone, either with or without
    opioids according to the comparison */

    MODEL (ta,tb)* DEATH(0) = LAOPIOID NEWOPIOID DOSE30TO59 DOSE60PLUS HYPOPIOID;
    /* SAS counting process syntax, where ta is the day prior to the current day of followup, tb is the current
    followup day, DEATH is the endpoint (either out-of-hospital or all deaths), and HYPOPIOID is the design
    variable*/
    /*  the variables LAOPIOID NEWOPIOID DOSE30TO59 DOSE60PLUS characterize the opioid exposure */

RUN;
```

Sensitivity Analyses

Clustering by region: variance adjustment. In this analysis, the 5 geographic regions of the U.S.[b] were considered as a clustering factor, that is, the outcomes for cohort members could be correlated within clusters. Thus, we modified the proportional hazards analysis to do robust variance estimation, as described by Donner and Klar.[15]

Clustering by region and control for region. In this analysis we controlled for factors not included in the Medicare data that may vary between regions, such as income or education. Thus, the model included a term for each of the regions.

Covariates fixed at baseline. In the primary analysis, the propensity score was updated at the time of each prescription fill. In this sensitivity analysis, the propensity score was fixed at baseline and the analysis was stratified by deciles of baseline propensity score. Opioid use and related variables updated for each day of followup.

Time-dependent propensity score weights. Because the propensity score deciles leave open the possibility of residual confounding, this sensitivity analysis utilized all of the information available in the propensity score. The proportional hazards regression was weighted by time-dependent matching weights,[16] which were updated at the time of each prescription fill during followup, with robust variance estimation to account for the dependencies induced by weighting. Matching weights, bounded by 0 and 1, produce estimates with lower variance than inverse probability/odds of treatment weights and in populations with good propensity score overlap between the treated/control groups the estimates are asymptotically equivalent to the ATT.[16] Opioid use and related variables were updated for each day of followup.

---

[b] West, southwest, midwest, southeast, northeast, as defined at https://www.nationalgeographic.org/maps/united-states-regions.

Propensity-score matched, covariates fixed at baseline. Each new user of trazodone was matched to one patient with benzodiazepine use and one patient with z-drug use with the greedy algorithm[12] with a caliper of 0.20. Robust variance estimation was used to account for matched sets. Opioid use and related variables were updated for each day of followup.

J. Additional Results

Appendix Table H. Adjusted hazard ratios for comparisons of study hypnotics, according to concurrent opioid use.

	Benzodiazepine vs Trazodone	z-Drugs vs Trazodone	Benzodiazepine vs z-Drugs
	HR (95% CI)	*HR (95% CI)*	*HR (95% CI)*
No Concurrent Opioid Use			
Out-of-hospital	0.99 (0.81-1.22)	0.96 (0.76-1.23)	0.98 (0.79-1.21)
All	0.95 (0.80-1.12)	0.87 (0.72-1.05)	1.06 (0.90-1.25)
Concurrent Opioid Use			
Out-of-hospital	3.02 (1.83-4.97)	1.98 (1.14-3.44)	1.40 (0.96-2.05)
All	2.21 (1.52-3.20)	1.65 (1.09-2.50)	1.25 (0.92-1.69)

K. Reference List

1. Buysse DJ. Insomnia. *JAMA.* 2013;309(7):706-716.
2. Winkelman JW. Clinical practice: Insomnia disorder. *N Engl J Med.* 2015;373(15):1437-1444.
3. Fagiolini A, Comandini A, Catena Dell'Osso M, Kasper S. Rediscovering trazodone for the treatment of major depressive disorder. *CNS Drugs.* 2012;26(12):1033-1049.
4. Von Korff M, Saunders K, Ray GT, et al. De facto long-term opioid therapy for noncancer pain. *Clin J Pain.* 2008;24:521-527.
5. Neutel CI, Johansen HL. Association between hypnotics use and increased mortality: causation or confounding? *Eur J Clin Pharmacol.* 2015;71(5):637-642.
6. Ray WA, Chung CP, Murray KT, Cooper WO, Hall K, Stein CM. Out-of-hospital mortality among patients receiving methadone for noncancer pain. *JAMA Intern Med.* 2015;175(3):420-427.
7. Ray WA, Chung CP, Murray KT, Hall K, Stein CM. Prescription of long-acting opioids and mortality in patients with chronic noncancer pain. *JAMA.* 2016;315:2415-2423.
8. Simard M, Sirois C, Candas B. Validation of the Combined Comorbidity Index of Charlson and Elixhauser to Predict 30-Day Mortality Across ICD-9 and ICD-10. *Med Care.* 2018;56(5):441-447.
9. Quan H, Sundararajan V, Halfon P, et al. Coding algorithms for defining comorbidities in ICD-9-CM and ICD-10 administrative data. *Med Care.* 2005;43(11):1130-1139.
10. Kim DH, Schneeweiss S, Glynn RJ, Lipsitz LA, Rockwood K, Avorn J. Measuring Frailty in Medicare Data: Development and Validation of a Claims-Based Frailty Index. *The journals of gerontology Series A, Biological sciences and medical sciences.* 2018;73(7):980-987.
11. Shadish WR, Steiner PM. A primer on propensity score analysis. *Newborn & Infant Nursing Reviews.* 2010;10(1):19-26.
12. Austin PC. An introduction to propensity score methods for reducing the effects of confounding on observational studies. *Multivariate Behav Res.* 2011;46:399-424.
13. Haukoos JS, Lewis RJ. The Propensity Score. *Jama.* 2015;314(15):1637-1638.
14. Ray WA, Liu Q, Shepherd BE. Performance of time-dependent propensity scores: a pharmacoepidemiology case study. *Pharmacoepidemiol Drug Saf.* 2015;24:98-106.
15. Donner A, Klar N. *Design and analysis of cluster randomization trials in health research.* 1st ed. London: Arnold; 2000.
16. Desai RJ, Franklin JM. Alternative approaches for confounding adjustment in observational studies using weighting based on the propensity score: a primer for practitioners. *Bmj.* 2019;367:l5657.
